# Transposable elements contribute to tissue-specific gene regulation in humans

**DOI:** 10.1007/s13258-024-01550-6

**Published:** 2024-08-01

**Authors:** Arsala Ali, Ping Liang

**Affiliations:** 1 Department of Biological Sciences, Brock University, St. Catharines, ON L2S 3A1 Canada; 2 Centre of Biotechnologies, Brock University, St. Catharines, ON L2S 3A1 Canada

**Keywords:** Transposable elements, Tissue-specific genes, Gene regulation, ENCODE

## Abstract

**Background:**

Transposable elements (TEs) contribute to approximately half of the human genome, and along with many other functions, they have been known to play a role in gene regulation in the genome. With TEs’ active/repressed states varying across tissue and cell types, they have the potential to regulate gene expression in a tissue-specific manner.

**Objective and methods:**

To provide a systematic analysis of TEs’ contribution in tissue-specific gene regulation, we examined the regulatory elements and genes in association with TE-derived regulatory sequences in 14 human cell lines belonging to 10 different tissue types using the functional genomics data from the ENCODE project. Specifically, we separately analyzed regulatory regions identified by three different approaches (DNase hypersensitive sites (DHS), histone active sites (HA), and histone repressive sites (HR)).

**Results:**

These regulatory regions showed to be distinct from each other by sharing less than 2.5% among all three types and more than 95% showed to be cell line-specific. Despite a lower total TE content overall than the genome average, each regulatory sequence type showed enrichment for one or two specific TE type(s): DHS for long terminal repeats (LTRs) and DNA transposons, HA for short interspersed nucleotide elements (SINEs), and HR for LTRs. In contrast, SINE was shown to be overrepresented in all three types of regulatory sequences located in gene-neighboring regions. TE-regulated genes were mostly shown to have cell line specific pattern, and tissue-specific genes (TSGs) showed higher usage of TE regulatory sequences in the tissue of their expression. While TEs in the regulatory sequences showed to be older than their genome-wide counterparts, younger TEs were shown to be more likely used in cell line specific regulatory sequences.

**Conclusions:**

Collectively, our study provided further evidence enforcing an important contribution of TEs to tissue-specific gene regulation in humans.

**Supplementary Information:**

The online version contains supplementary material available at 10.1007/s13258-024-01550-6.

## Introduction

Transposable elements (TEs), also known as mobile elements (MEs), are interspersed repeats constituting a major portion of the genomes of animals and plants (Koning et al. 2011; SanMiguel et al. 1996). TEs have important implications in the genome structure and function including insertion- and recombination-based structural variants, generation of microsatellites, and creation of new genes through molecular domestication events (Ayarpadikannan and Kim 2014; Cordaux and Batzer 2009; Balachandran et al. 2022; Etchegaray et al. 2022; Zattera and Bruschi 2022). Among these, an important function of TEs in the genome is their role in gene regulation. In this regard, TEs have intrinsic regulatory properties for providing *cis* acting regulatory sequences (Swergold 1990; Roy et al. 2000; Regenmortel and Mahy 2010; Jacques et al. 2013). By harboring binding sites for a wide range of *trans*-acting and chromatin remodeling factors, TEs have a versatile role in gene regulation by providing both positive and negative regulatory elements in the genome. Some of such examples include an Alu-derived enhancer in *CD8* gene (Hambor et al. 1993), a L1-derived alternative promoter of *CHRM3* gene (Kim and Hahn 2011), and epigenetic silencing of L1s leading to downregulation of nearby genes (Liu et al. 2018). TE-derived regulatory sites are found to be conserved as well as lineage-specific involving both old and young TEs. As examples, a SINE-derived promoter of *POMC* gene was exapted before the origin of *Prototherians* (Franchini et al. 2011), while evolutionarily young and hominid-specific TEs belonging to HERVK and HERVH are found to act as enhancers in human embryonic stem cells and during gastrulation and fetal development (Pontis et al. 2019, 2022).

Regulatory elements in the genome harbor characteristic chromatin structures and chromatin modification signatures defined by chromatin accessibility and specific histone marks. Some of the histone modifications are widely considered as activating marks, such as H3K4me3 and H3K27ac (Wysocka et al. 2006; Creyghton et al. 2010), while certain other histone modifications (e.g., H3K27me3 and H3K9me3) are known to broadly associate with chromatin condensation and transcription repression (D’Urso and Brickner 2014; Hublitz et al. 2009). Identification of open chromatin sites and histone modification markers are therefore important targets in large-scale projects like ENCODE (Feingold et al. 2004) to provide comprehensive maps of candidate regulatory regions in the human genome. Several genome-wide studies have analyzed the contribution of TEs in these regulatory regions and showed that a large fraction of these regions involve TEs from multiple families showing enrichment in active and repressed chromatin regions, implying a widespread role of TEs in gene regulation (Jacques et al. 2013; Trizzino et al. 2018).

TEs’ active/repressed epigenetic states differ across tissue types (Trizzino et al. 2018; Jiang et al. 2024), and TE-derived regulatory sites thus tend to be tissue-specific making them as crucial players in tissue-specific gene regulation. Genes associated with TEs in active regulatory chromatin regions have been shown to have higher expression variance across tissues compared to genes not containing TEs in active regulatory chromatin regions (Trizzino et al. 2018). Furthermore, a few gene-specific studies have experimentally identified tissue-specific promoters/enhancers derived from TEs. For examples, two TE-derived neuronal enhancers are shown to regulate *POMC* gene expression in neurons (Franchini et al. 2011), and a TE-derived T-cell specific promoter and an intronic enhancer are shown to induce T-cell expression of *FCER1G* and *CD8*, respectively (Hambor et al. 1993; Brini et al. 1993). Tissue-specificity of TEs’ active/repressed states (Trizzino et al. 2018) and identification of TE-derived tissue-specific promoters by these gene-specific studies (Hambor et al. 1993; Franchini et al. 2011; Brini et al. 1993) motivated systematic studies of TE-regulated genes across different tissue types. Two studies (Nikitin et al. 2019; Igolkina et al. 2019) determined TE-regulated genes in the human genome using regulatory sequence annotation data (particularly TF ChIP-seq and histone ChIP-seq data) of multiple cell lines from different tissues. In these studies, genes were scored based on TE content in the neighbouring regulatory region and the top-ranking genes were inferred as being TE-regulated. Immune response and carbohydrate and fatty acid metabolism were among the major processes found enriched by TE regulation. Deducing TE-regulated genes in a genome-wide manner, the work provides useful insight following gene-level studies. However, in these studies genes were ranked based on average TE enrichment score in different cell lines disregarding the tissue-specific component. With variation in TEs’ active states across tissues being reported (Trizzino et al. 2018; Jiang et al. 2024), it should be more meaningful to investigate TE regulation of genes in a tissue-specific manner. With this in mind, our study primarily aims to infer and compare TE-regulated genes in 14 cell lines for 10 tissues using a systemic approach based on datasets representing three types of regulatory regions including DNase hypersensitive sites, histone active sites, and histone repressive sites. We found that more than 95% of the regulatory regions were cell line specific and with a higher fraction being TE-derived compared to that of shared regulatory regions, and majority of the potentially TE-regulated genes are cell line specific with cases showing functions relevant or specific to the tissue of origin, all highlighting the contribution of TEs in tissue-specific gene regulation.

## Materials and methods

### Selection of datasets for gene regulatory sequences

For our study, we chose to use the functional genomics datasets generated by ENCODE (Feingold et al. 2004), specifically, the regulatory sequence datasets generated using three methods including, DNase-seq experiment, ChIP-seq experiment for active histone marks (H3K4me3, H3K27ac, H3K9ac, H3K79me2), and ChIP-seq experiment for repressive histone marks (H3K27me3, H3K9me3). These methods demarcate DNase hypersensitive sites (DHS), histone active sites (HA) and histone repressive sites (HR), respectively. A total of 14 cell lines were selected for covering all three types of regulatory sites without flags for issues (e.g., extremely low read depth). These cell lines include DND-41 (blood, T-lineage), GM12878, Karpas-422, MM.1S and NCIH929 (blood, B-lineage), SK-N-SH (brain), MCF-7 (breast), HeLa-S3 (cervix), HCT116 (colon), HepG2 (liver), IMR-90 (lung), PC-9 (lung), PC-3 (prostate), and GM23248 (skin). For each of these cell lines, we retrieved from the ENCODE data portal the narrowpeak.bed files, which provide the genomic coordinates of the signal peaks in the GRCh38 reference human genome, with the cell line name, tissue of origin and file names for each data type detailed in Table S1.

The genomic coordinates of TE positions based on RepeatMasker (Nishimura 2000) annotation for human GRCh38 reference genome were retrieved from UCSC genome browser website (Karolchik 2003). The complete list of human-specific transposable element/mobile element insertions (HSME) with coordinates based on GRCh38 assembly was retrieved from the data deposition by Tang and Liang (2019). The transcript IDs for the principal transcript isoforms of protein-coding genes were retrieved from APPRIS (Rodriguez et al. 2015), while their transcription start sites (TSSs) were retrieved from GENCODE (the basic gene annotation file) (Harrow et al. 2012). A catalog of tissue-specific genes (TSGs) by expression for the 10 tissues associated with the 14 cell lines used was retrieved from ‘The Human Protein Atlas’ database (Thul and Lindskog 2018).

### Analysis of TE composition and age profile in regulatory regions

For each of the three datasets (DHS, HA, HR), the regulatory regions from all 14 cell lines were merged as a set of non-overlapping regions in the genome, which was next categorized into cell line specific (defined as present in one or more but not all of the 14 cell lines) and shared regions (present in all 14 cell lines), as well as gene-neighboring (10 Kb surrounding TSS, i.e., 5 Kb on either side of TSS) and genome-wide regions (all identified regulatory regions) for comparative analysis. Further, we compared TE composition (by four main TE types) to see whether there is overrepresentation and/or underrepresentation of TE types between (1) whole genome and regulatory regions, (2) cell line specific and shared regulatory regions, and (3) gene-neighboring and genome-wide regulatory regions. In addition, using sequence divergence of individual TEs from their consensus sequences as an estimate of their insertion age, we compared the age profile of TE types between the same three pairs as the last step.

### Determination of correlation and clustering among cell lines based on TE profile of genes’ regulatory region

For each of the 19,674 protein-coding genes, we determined TE density of their neighboring regulatory region in every cell line as the fraction of gene-neighboring regulatory region being TE-derived (i.e., length of TE-derived regulatory region in gene-neighboring site / length of total regulatory region in gene-neighboring site). Based on the regulatory region TE density of all genes, correlation was determined between all possible pairs of the 14 cell lines.

### Identification of genes enriched with TE-derived regulatory sites

To identify genes enriched with TE-derived regulatory sites, we collected for every cell line the genes with regulatory region having TE density ≥ 0.1, (i.e., ≥ 10% of neighboring regulatory region being TE-derived) for DHS and HR datasets. For HA, the cutoff was increased to ≥ 0.3, since for HA regions genes have a higher average regulatory region TE density. The lists of genes were then subjected to enrichment analysis with Toppcluster (Kaimal et al. 2010) to compare enriched biological processes for the potential TE-regulated genes in each cell line. Specifically, following the program’s specification for input, a two-column list of genes with first column being gene names and second column being cluster (cell line) names was input into the Toppcluster web server. Functional enrichment was selected as analysis parameter and Benferroni correction was used with *p* value cutoff as 0.05.

### Analysis of TSGs

For each of the 10 tissues involved in the study (blood (B-lineage), blood (T-lineage), brain, breast, cervix, colon, liver lung, prostate, skin), a list of TSGs by expression was retrieved from ‘The Human Protein Atlas’ database (Thul and Lindskog 2018) and their average TE density of the regulatory regions in the respective tissue was compared to that in other tissues. Moreover, for every tissue type, we compared the fraction of TSGs enriched for TE-derived regulatory sites in the respective tissue versus other tissues. Further, TSGs that harbor TE-derived regulatory sites only in the respective tissue (i.e. regulatory region TE density ≥ 0.1 in the respective tissue and being 0 in all other tissues) were also catalogued.

### Statistical and computational analysis

The statistical tests used in the study include (1) Chi-square test for calling overrepresentation or underrepresentation of TE types in the regulatory regions, (2) Pearson’s correlation test for testing pairwise correlation between cell lines based on the regulatory region TE density of all protein-coding genes, and (3) two tailed T-test for comparing the average sequence divergence of TEs across different types of regulatory regions and for comparing the average TE density of the regulatory regions of TSGs between respective tissue and other tissue types. These tests were performed using software/tools including R, MedCalc, and GraphPad Prism, while plots were generated using a combination of R, MS excel, and GraphPad Prism. Most of the computational analysis was performed using a combination of bedtools (Quinlan and Hall 2010) (for merging, intersecting, and subtracting genomic regions among lists), awk utility, and Linux shell scripts on the high-performance computing servers provided by Digital Research Alliance of Canada.

## Results

**Fig. 1:**
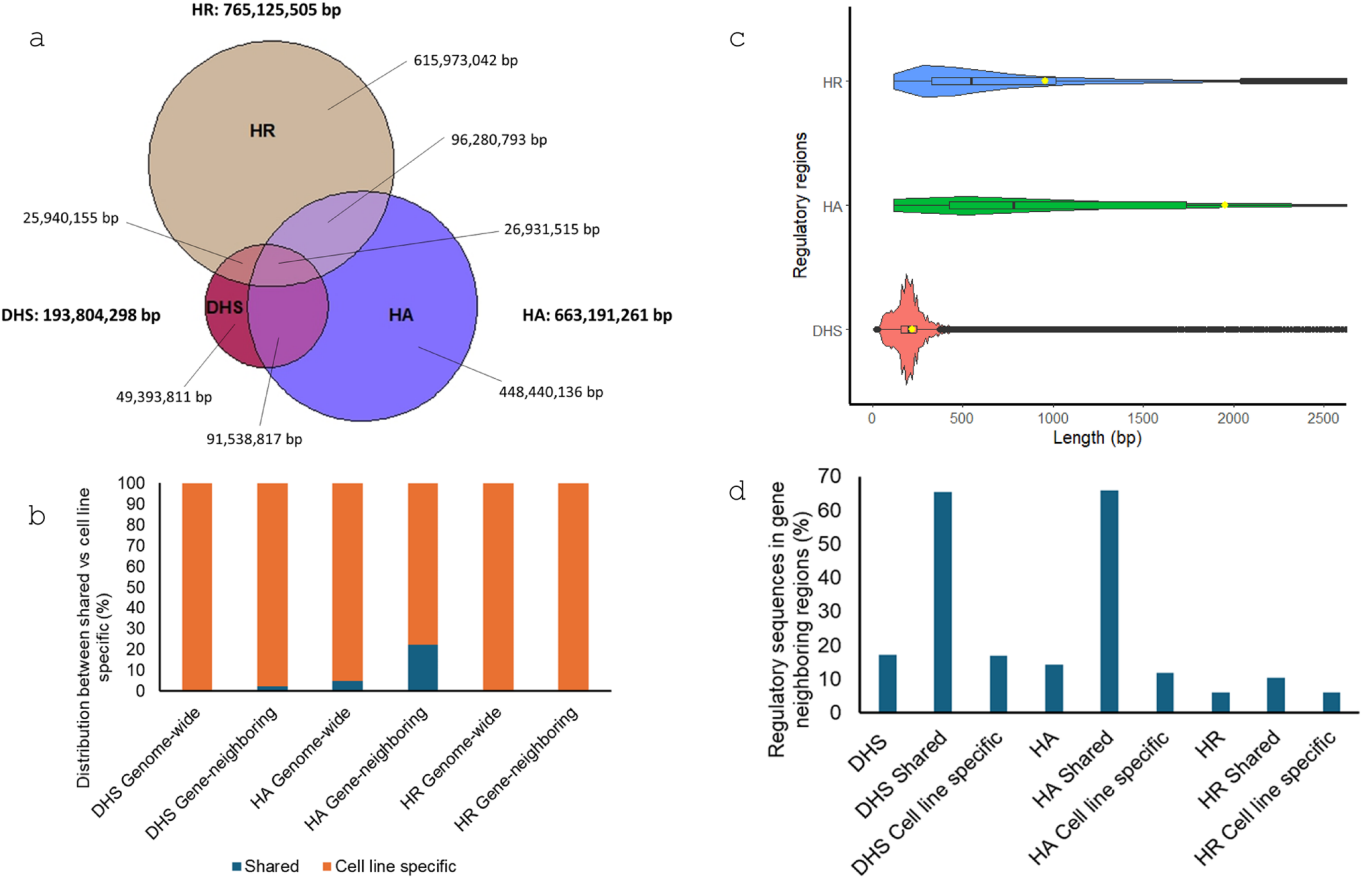
Patterns of regulatory sequences from ENCODE represented as DNase hypersensitive sites (DHS), histone active sites (HA) and histone repressive sites (HR) in 14 cell lines. **a** Overlap among the three regulatory region datasets; **b** Proportion of regulatory regions being cell line specific and shared (shared defined being common to all 14 cell lines). Negligible proportion is shared for HR; **c** Length distribution of DHS, HA and HR after merging the peak intervals of all cell lines (the yellow dot shows the average); **d** Proportion of regulatory regions as gene-neighboring

In this study, we aimed to analyze TEs’ contribution to gene regulation in the human genome with a focus on tissue-specific regulation and the characteristics of the associated genes. For this purpose, we analyzed three regulatory sequence datasets including DNase-seq peaks, histone ChIP-seq peaks for active histone marks and histone ChIP-seq peaks of repressive histone marks. Specifically, we collected ENCODE data files for 14 cell lines, for which all three types of regulatory data types are available, and these cell lines cover 10 different organs/tissues (Table S1). For each of the three types of regulatory sequences, the regions for all 14 cell lines were merged as a list of non-overlapping regions. Interestingly, the three types of regulatory sequences showed to be mostly distinct from each other with only less than 2.5% being shared among all three (Fig. 1a), indicating that each dataset represents a unique type of regulatory sequences in the human genome. For this reason, we analyzed each of the three datasets separately, instead of combining them as one list, to better understand their unique aspects regarding cell line specificity, TE composition and age profile, as well as characteristics of genes with regulatory sequences containing high levels of TEs.

### Most regulatory regions are cell line specific

At the top level, among the three types of regulatory sequences, DHS is more than 10 times shorter by total sequence length than that of HA and HR, and proportionally, DHS and HA share more in common than between DHS vs HR and HA vs HR (Table 1, Fig. 1a). This is expected as DHS and HA both represent active regulatory sequences, while HR represent negative regulation. With DHS and HA being active regulatory sequences captured by different techniques, we were also interested in examining the differences between the two data types based on their overlap with RNAPII binding sites, transcription factor binding sites (TFBSs) and putative promoter regions (1.5 Kb upstream of TSS). Interestingly, among the three regulatory data sets, DHS showed the higher fraction being overlapped with these additional regulatory datasets (Table S11).

**Table 1:** Composition of transposable elements by types in different regulatory regions

Regulatory region	Total regulatory sequences (bp)	regions (#)	Average length (bp)	Total TE length (bp)	TEs (%)	TE fragments (#)	Total SINE length (bp)	Total LINE length (bp)	Total LTR length (bp)	Total DNA length (bp)
*DHS*										
Overall	193,804,298	890,289	218	59,781,406	31	494,992	13,779,108	22,085,632	17,056,469	6,860,197
Shared	1,228,843	11,484	107	56,049	5	903	7,120	14,623	24,549	9,757
Cell line specific	192,575,455	901,750	214	59,725,357	31	494,961	13,771,988	22,071,009	17,031,920	6,850,440
*HA*										
Overall	663,191,261	339,844	1,951	264,348,816	40	1,152,523	101,894,646	97,069,953	41,300,573	24,083,644
Shared	32,250,645	46,594	692	9,301,264	29	57,092	5,053,518	2,616,564	762,119	869,063
Cell line specific	630,940,616	386,438	1,633	255,047,552	40	1,123,122	96,841,128	94,453,389	40,538,454	23,214,581
*HR*										
Overall	765,125,505	800,974	955	355,284,601	46	1,362,462	66,853,854	166,094,678	97,316,793	25,019,276
Shared	2,819	12	235	647	23	3	124	-	523	-
Cell line specific	765,122,686	800,986	955	355,283,954	46	1,362,462	66,853,730	166,094,678	97,316,270	25,019,276

For DHS regions, a total of 193,804,298 bp was identified in 890,289 non-overlapping regions, out of which only 1,228,843 bp (0.6%) for 11,484 regions are shared by all cell lines and the rest 99.4% are considered cell line specific (present in one or more but not all cell lines). For HA, there is a total of 663,191,261 bp sequence in 339,844 regions with 630,940,616 bp (95.1%) for 386,438 regions being cell line specific, while for HR, there is a total of 765,125,505 bp sequence for 800,974 regions with almost all (> 99.9%) being cell line specific (Table 1, Fig. 1a, b). Therefore, all three types of regulatory sequences showed to be mostly cell line specific with HR sequences having the highest ratio being cell line specific, while HA sequences had slightly lower rate for being cell line specific. It is interesting to notice that by average length, HA sequences are about two times longer than that of HR (1,951 bp vs 955 bp), while DHS are the shortest being 218 bp or ~ 1/9 of that for HA (Table 1, Fig. 1c). Furthermore, for all three types, the cell line specific sequences are at least 2 times longer by average length than the shared ones with HR showing the largest discrepancy (~ 4 times) (Table 1), indicating the unique nature of regulatory sequences by functional type and by cell line specificity.

Further, we examined the distribution of these regulatory sequences in the gene-neighboring region (10 Kb surrounding TSS). The proportion of DHS, HA and HR in the gene-neighboring region is 17.3%, 14.4% and 6.1% with 33,441,184 bp (120,296 regions), 95,394,393 bp (32,402 regions), and 46,897,183 bp (42,329 regions), respectively (Table 2). Therefore, DHS has the highest proportion being gene-neighboring (17.3%), followed by HA (14.4%) being lower and HR being much lower (6.1%). The shared regulatory regions showed a much higher ratio being gene-neighboring than the cell line specific regions with HA having the highest (66.0%), followed by DHS (65.5%), and HR having the lowest (10.3%) (Fig. 1d). On the other hand, like for their counterparts in the whole genome, the ratio of gene-neighboring regulatory sequences being cell line specific is very high, with that for HR being the highest (100%), followed by DHS (97.6%) and HA (77.7%) (Fig. 1c). In this case, the ratio of being cell line specific for HA dropped from 95.1% for genome-wide to 77.7% for gene-neighboring regions (Fig. 1c). For all three types, the average length of regulatory sequences showed noticeable increase from the genome-wide counterpart (Tables 1, 2).

**Table 2:** Composition of transposable elements by types in gene-neighboring regulatory stes (10 Kb surrounding TSS)

Regulatory region in gene-neighboring site	Total regulatory sequences (bp)	regions (#)	Average length (bp)	Total regulatory TE (bp)	TEs (%)	TE fragments (#)	Total SINE (bp)	Total LINE (bp)	Total LTR length (bp)	Total DNA length (bp)
*DHS*										
Overall	33,441,184	120,296	278	5,449,514	16	50,086	2,299,904	1,605,673	1,006,559	537,378
Shared	805,447	7,240	111	9,403	1	161	2,797	1,568	3,718	1,320
Cell line specific	32,635,737	127,514	256	5,440,111	17	50,079	2,297,107	1,604,105	1,002,841	536,058
*HA*										
Overall	95,394,393	32,402	2,944	28,272,410	30	141,400	15,594,689	7,295,908	3,090,770	2,291,043
Shared	21,275,627	22,416	949	4,382,823	21	27,424	2,599,860	1,127,314	276,003	379,646
Cell line specific	74,118,766	53,792	1,378	23,889,587	32	124,456	12,994,829	6,168,594	2,814,767	1,911,397
*HR*										
Overall	46,897,183	42,329	1,108	13,383,750	29	68,896	4,949,076	4,756,034	2,657,062	1,021,578
Shared	290	2	145	–	–	–	–	–	–	–
Cell line specific	46,896,893	42,331	1,108	13,383,750	29	68,896	4,949,076	4,756,034	2,657,062	1,021,578

Overall, DHS, HA, and HR regulatory sequences mostly showed to be a cell line specific with a considerable portion locating into the gene-neighboring regions and each showed to be mostly unique group of regulatory sequences in the human genome by locations, average size, and rate being cell line specific.

### Different types of regulatory sequences showed different pattern of TE enrichment

**Fig. 2:**
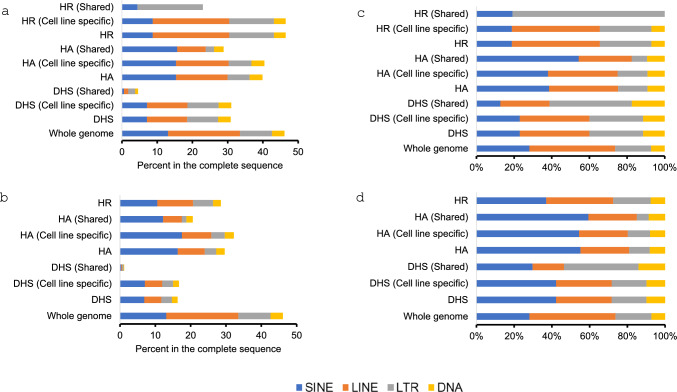
TE composition in the genome-wide and gene-neighboring regulatory regions in comparison with the whole genome. **a** and **c** Comparison of TE composition in the genome-wide DHS, HA, HR vs to the whole genome, showing percentage of different TE types in the regulatory sequence (**a**) and percentage of TE types in the TE regulatory sequences (**c**)**; b** and** d **TE composition in the gene-neighboring (10 Kb surrounding TSS) DHS, HA and HR in comparison to the whole genome, showing percentage of different TE types in the regulatory sequence (**b**) and percentage of TE types in the TE regulatory sequences (**d**). Please note that there is no data available for shared HR as almost all of the gene-neighboring HR region is cell line-specific

To assess TEs’ contribution in the regulatory sequences described above, we examined their TE composition. Overall, TEs contribute to at least one third of these 3 types of regulatory sequences with the rate being 30.8%, 39.9%, and 46.4% for DHS, HA, and HR, respectively (Table 1, Fig. 2a). In contrast, the rate of TEs in the gene-neighbouring regulatory sequences is much lower than their genome-wide counterpart (all regulatory sequences for a type), while still being significant at 16.3%, 29.6%, and 28.5% in the DHS, HA, and HR regions, respectively (Table 2, Fig. 2b). It is worth noting here that the overall rates of TEs in the regulatory regions are lower than the proportion of TEs in the genome, which is ~ 48% (bottom bars in Fig. 2a,b) (Lander et al. 2001; Tang et al. 2018), indicating an overall pattern of TE de-enrichment in these regulatory sequences, among which a higher degree of de-enrichment of TEs is seen in DHS and HA than HR. Still, we examined to see if there is any relative overrepresentation among TE types in these regulatory sequences in comparison to that in the genome based on the relative percentage across the TE types. In this regard, DHS showed an overrepresentation of LTRs (29% vs 19%) and DNA transposons (11% vs 7%), and such overrepresentation is even much higher in shared DHS for being 44% (LTR) and 17% (DNA), respectively (Fig. 2c). In HA, SINE is the most overrepresented TE class (39% vs 28%) overall and more so in the shared regions (59%), while in HR, LTR is the most overrepresented TE class (27% vs. 19%) overall, and as high as 80% in the shared regions (Fig. 2c). In all these cases, the over-representation over the whole genome is statistically significant (the Pearson’s chi squared test *p* < 0.0001). Interestingly, the same analysis for the gene-neighbouring regulatory sequences showed a very different profile with SINEs being enriched in all three types of regulatory sequences for being 42% in DHS, 55% in HA, and 37% in HR vs 28% in the genome (Pearson’s chi squared test: *p* < 0.0001) (Fig. 2d).

Overall, while all three types of regulatory sequences showed a lower level of total TE content in comparison to the genome average, by relative ratio among TE types, each showed enrichment for one or two specific TE type(s); specifically, DHS for LTRs and DNA transposons, HA for SINEs, and HR for LTRs. In contrast, SINEs seem to be the only TE class overrepresented in all three types of regulatory sequences located in gene-neighboring regions.

### TEs show unique age profiles in different types of regulatory sequences

**Fig. 3:**
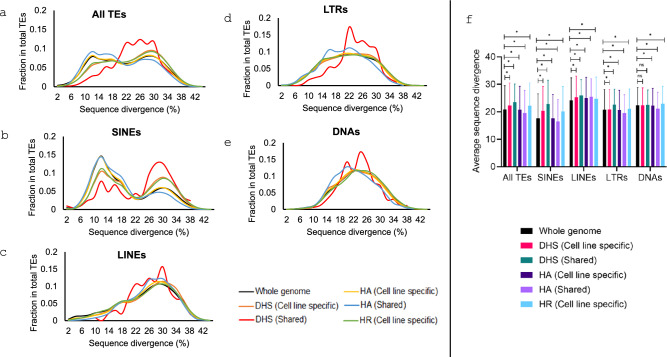
Age profile comparison between TEs in the regulatory regions and those in the whole genome. **a**–**e** Line graph showing fraction of total TEs in different age classes (using sequence divergence from their consensus sequences as estimate of age) in cell line specific and shared regulatory regions in comparison to the whole genome; **f** Average sequence divergence of TEs in cell line specific and shared regulatory regions compared to the whole genome

We examined the age profiles of TEs involved in the regulatory sequences in comparison with that of the whole genome and across types of regulatory sequences and TEs. The TE age is determined based on the sequence divergence of individual TEs from their perspective consensus sequences. As shown in Fig. 3, across types of regulatory sequences, TEs in shared DHS seem to have older age profiles significantly deviated from that in the whole genome, to which the TE age profiles of other regulatory sequence are more similar (Fig. 3a). In case of DHS, all TE types showed higher ages in shared regulatory region compared to cell line specific regulatory regions (Fig. 3). Intrigued by observing younger TEs in cell line specific regulatory regions, we also examined the ratio of human-specific TEs to all TE ratio in cell line specific versus shared regulatory region and found the ratio to be higher for cell line specific regulatory regions (Table S12). Across TE types, quite different age profiles are observed with SINEs showing a bi-modular profile with one peak at a much lower divergence (i.e., younger age, Fig. 3b), while all other three TE types showing basically a mono-modular distribution at older ages (Fig. 3c–e). This matches what we know about the unique temporal proliferation profiles of these TE classes during primate evolution (Kramerov and Vassetzky 2011). The profile of SINEs in shared DHS shows a clear deviation from other regulatory sequences by having a much higher older peak and a lower younger peak (Fig. 3b), indicating their older average age of SINEs among all categories of regulatory sequences. The age difference of TEs between different groups of regulatory sequences is mostly statistically significant (Fig. 3f). This difference is even more dramatic for TEs in gene-neighboring regulatory sequences (Fig. S1).

**Fig. 4:**
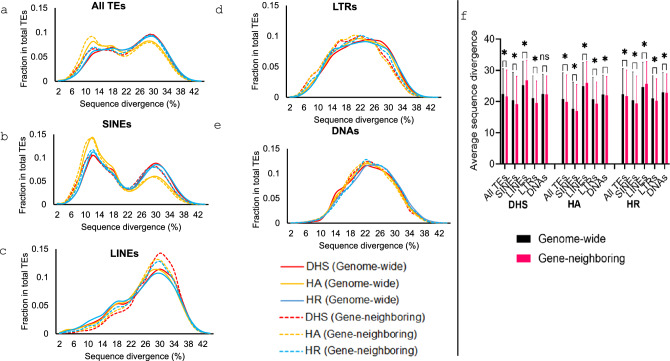
Age profile comparison between TEs in all regulatory regions and those in gene-neighboring regions. **a**–**e** Line graph showing fraction TEs in total TEs at different sequence divergence levels; **f** Average sequence divergence of TEs in genome-wide regulatory regions compared to gene-neighboring regulatory regions

A few additional interesting notes can be made about TE age profiles. First, LINEs tend to distribute more towards older ages in the regulatory regions compared to whole genome, more for those in gene-neighbouring regions (this is so much so have for those in the shared DHS regions (Fig. 3c and S1c). This can also be observed in pairwise average TE age comparison between whole genome and each type of regulatory regions within the same TE class (Fig. 3f and S1f). Second, while SINEs, LTRs, and DNAs tend to be older in genome-wide regulatory regions compared to gene-neighboring regulatory regions, LINEs show the opposite pattern (Fig. 4). For example, the average sequence divergence of SINEs and LINEs in genome-wide vs gene-neighboring DHS is 20.5 vs 19.2 and 25.2 vs 26.8, respectively, and the difference is statistically significant for most comparisons (Two-tailed t test: *p* < 0.0001) (Fig. 4f).

### Weak to moderate correlation between cell lines based on TE profile of gene-neighbouring regulatory regions

TE density of the gene-neighboring regulatory regions was determined for every protein-coding gene in each of the cell lines as a basis to analyze the degree of correlation between cell lines. There seemed to be no strong correlation between cell lines with most showing negligible or low correlation (Pearson correlation coefficient, r = 0–0.5) in case of DHS and HR, while for HA, moderate to strong correlation was observed for most of the pairwise comparisons (Pearson correlation coefficient, r = 0.5–0.75) (Fig. S2, Table S2). As shown in Fig. S2, some meaningful clustering of cell lines by tissue type was observed. For examples, the 5 cell lines from blood showed a distinct clustering by TE density in HA and to a less clean clustering by TE density in DHS and HR.

### TE-regulated genes are mostly cell line specific by expression

**Fig. 5:**
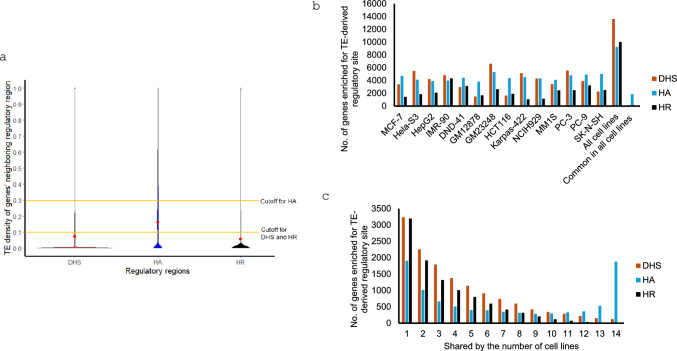
TE density of genes’ regulatory region and genes enriched with TE-derived regulatory sites. **a** Violin plots representing the distribution of regulatory TE density of protein-coding genes in all 14 cell lines (red dot indicates the average regulatory region TE density of genes, yellow line shows the cutoff to collect genes enriched with TE-derived regulatory sites); **b** Number of genes enriched for TE-derived regulatory sites in different cell lines; **c** Cell line specificity of the genes enriched with TE-derived regulatory sites. The plot shows the number of potential TE-regulated genes that are specific to only one cell line and the number shared by 2, 3 and as many as all 14 cell lines

To find if there are any observable patterns among TE-regulated genes, defined as protein-coding genes with TE density in the neighboring regulatory regions ≥ 10% (or ≥ 30% for HA, chosen based on distribution pattern as seen Fig. 5a) were identified and analyzed for enrichment of gene ontology (GO) terms for biological processes and compared among cell lines. Different cell lines show different enriched GO terms with many common to some but not all cell lines. Importantly, in multiple cases, the enriched biological processes are relevant to the particular tissue type from which the genes were identified with TE-enriched regulatory sequences (Tables 3, S6–S8). In other words, genes showing TE-regulation in a tissue tend to have functions related to the tissue, suggesting contribution of TEs to tissue-specific functionalities. Some interesting examples for each of the three types of regulatory sequences are described below, with a few additional ones mentioned in the discussion section.

**Table 3:**
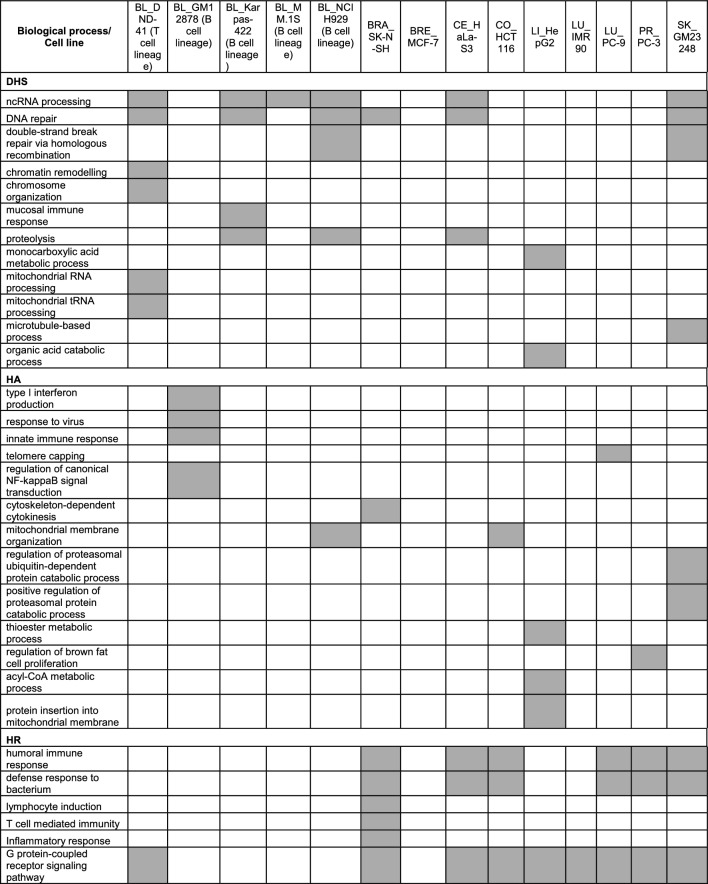
Enriched biological processes among potential TE-regulated genes in different cell lines*

*Highlighted cells show biological processes enriched in TE-regulated genes in the respective cell line

*DHS*: In all 14 cell lines a total of 13,619 TE-regulated genes were collected, among which 3,239 genes were specific to only one cell line and 127 genes were common to all 14 cell lines (Fig. 5b, c). Table 3 and S6 provide comparison among cell lines for the enriched biological processes GO terms with some relevant to tissue-specific functions. For examples, immune response processes were found enriched for blood cell line (Karpas-422). Furthermore, some biological processes related to response to stress/DNA damage were found enriched only for blood cell lines (NCIH929, Karpas-422) and skin cell line (GM23248), which have been reported as vulnerable to external environment and challengeable by intrinsic and exogenous stress (Hu et al. 2018; Markiewicz and Idowu 2019). Besides, some GO terms related to chromatin assembly and organization were enriched only in blood T-lineage cell line (DND-41). Interestingly, multi-level chromatin remodelling has been reported to be involved in human T-cell activation (Bediaga et al. 2021) (Tables 3, S6).

*HA*: In all 14 cell lines a total of 9242 TE-regulated genes were collected, among which 1,906 genes were specific to only one cell line and 1,882 genes were common to all 14 cell lines, with the latter being more than 10 times higher than that of DHS, more so if by ratio (Fig. 5b, c). Some biological processes including mitotic cell cycle and nucleocytoplasmic transport were found enriched in all 14 cell lines, however, multiple biological processes were found enriched for one or more but not all 14 cell lines. Tables 3 and S7 provide comparison among cell lines for the enriched biological processes GO terms, with some associated with tissue-specific functions. As examples, GO terms related to immune related processes (cytokine production, response to virus, innate immune response) and NF-kappaB signaling regulation, which has a vital role in lymphocyte development and function (Gerondakis and Siebenlist 2010), were found enriched for blood cell line (GM12878). Further, some GO terms for positive regulation of proteosomal ubiquitin-dependent processes, which have been implicated in regulating skin pigmentation (Ando et al. 2009), were found enriched only for skin cell line (GM23248) (Tables 3, S7).

*HR*: Contrary to DHS and HA, HR represent negative regulatory elements potentially downregulating the genes. In all 14 cell lines a total of 10,021 TE-regulated genes were identified, among which 3,195 genes were associated with only one cell line (Fig. 5). These 10,021 genes showed enrichment in cell line-specific patterns with a few relating to tissue-specific functionalities (Tables 3, S8). Opposite to the pattern for genes associated with HA, biological processes related to immune/defense and lymphocyte activation response were not found enriched for any of the blood cell lines. Moreover, GPCR signaling processes were not found enriched for most of the blood cell lines, likely due to the cruciality of GPCR mediated signaling in lymphocyte function (Kehrl 2004) (Tables 3, S8).

### TSGs with tissue-specific TE-derived active regulatory regions

**Fig. 6:**
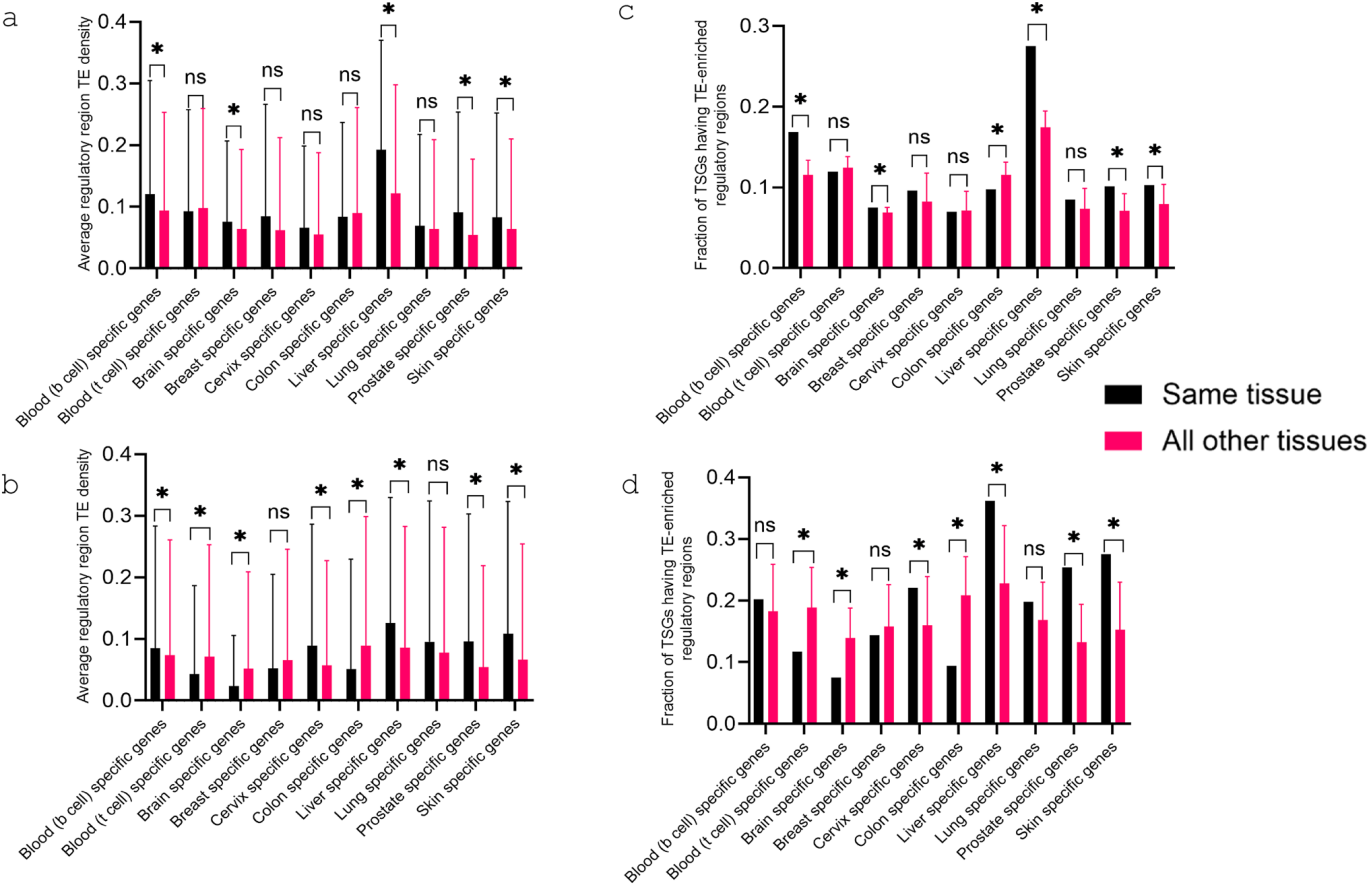
TE-derived active regulatory sites in tissue-specific genes (TSGs, by expression). **a** and** b** Average ‘regulatory region TE-density’ of the tissue-specific genes in the respective tissue and other tissues for DHS (**a**) and for HA (**b**); **c** and **d** Fraction of tissue-specific genes harboring TE-enriched regulatory region in the respective tissue and other tissues for DHS (**c**) and for HA (**d**)

To further examine the pattern of TE-derived regulatory sequences, we compared the TE density in the regulatory sequences of TSGs in the respective tissue versus all other tissue types. It was found that TSGs tend to have higher TE density in the active regulatory sequences in association with the tissue of their expression than those in other tissue types. For DHS, this was found to be the case for 8 of the 10 tissues covered in study with the difference being significant for 5 tissues, while for HA dataset, this was found to be true for 6 of the 10 tissues with the difference being significant for 5 tissues. Colon and blood (T lineage) did not show this trend with either DHS or HA (Fig. 6a, b). We also compared the ratio of TSGs identified as TE-upregulated in association with the tissue of their expression to that of those in other tissues. In this case, 7 of the 10 tissues showed higher rates of TE-regulated TSGs with the difference being significant for 6 tissues for both DHS and HA. Again, only blood (T lineage) did not show this trend with either DHS or HA (Fig. 6c, d). Furthermore, we identified the TSGs that are tissue-specific TE-regulated genes by having TE density of gene-neighboring regulatory site ≥ 10% in the respective tissue and no TE in their regulatory sites in all other tissues (see Table 4 for the complete listing of these genes and Tables S9 and S10 for these genes along with TE density value for every cell line). As interesting examples, *CYP4F3* gene (cytochrome P450) has more than 25% TE in gene-neighboring DHS from liver cell line (HepG2) but no TE-derived gene-neighboring DHS from any of the other cell lines; *OTC* (Ornithine decarboxylase) gene has 100% TE in gene-neighboring DHS only in liver cell line (HepG2). Similarly, *KRT82* (keratin 82) and *KRT72* (keratin 72) genes have > 30% gene-neighboring DHS being TE-derived only in skin cell line (GM23248), while *CD180* gene (Fc receptor) has more than 30% of gene-neighboring HA being TE-derived only in cell lines of B-cell lineage. These cases provided evidence for a direct link between TE-derived active regulatory sequences and the specific expression of genes in the respective tissue.

**Table 4:** Tissue-specific genes with tissue-specific TE-derived active regulatory sites

Tissue	Tissue-specific genes with TE density ≥ 10% in cell line(s) of the same tissue type and TE density = 0 in cell lines of all other tissue types under study	
	DHS	HA
Blood (B cell lineage)	ABO, ADD2, ANKRD24, BANK1, BRS3, CCR6, CHI3L2, CLCN4, CORO2B, DENND5B, FCER2, FCGR2B, FCRL2, FCRL5, FXYD7, GNG7, HLA-DMB, HLA-DRA, HLA-DRB1, HLA-DRB5, HSD17B14, IKZF3, IL5, IRF4, KLHL32, MAGEE1, MMP11, MS4A1, NBPF15, OR2AE1, OR3A1, OXCT2, PLA2G2D, PLEKHG7, PNOC, POU2AF1, PPP1R2C, SCN3A, SH2B2, SLC9C1, SPACA3, STK33, TNFRSF13C, TNFRSF17, TSPAN33, UCHL1, WDFY4, ZPBP2	ADAMDEC1, ADD2, AFF3, AMPD1, ANKRD24, BTLA, CCR6, CD180, CD80, CD86, CLEC17A, COL19A1, FCER2, FCGR2B, FCRL1, FCRL2, FCRL3, FCRL5, FGD2, GH1, GLYATL1B, GPR25, GRAP, HLA-DQA1, HLA-DQB1, HLA-DRA, HLA-DRB5, HTR3A, IKZF3, IRF4, IRF5, KHDRBS2, KLHL32, LY86, MAGEE1, MEF2C, MS4A1, NLRP4, NPIPB6, OOEP, OR2T2, OXCT2, PEBP4, PLA2G2D, PLEKHG1, PPP1R2C, PVRIG, SCIMP, SCN3A, SIGLEC6, SLC9C1, SPACA3, SPATA48, TAL2, TCL1A, TLR10, TNFRSF13C, TNFRSF17, UGT2B17, WDFY4, ZNF98, ZPBP2
Blood (T cell lineage)	ADAMTS1, AQP7, C20orf204, CD2, CD40LG, CFH, GIMAP7, ITGA6, PRKCQ, TRPC3, YPEL4	CACNA2D4, CD2, CD247, CD8A, CUZD1, CXCR6, DNTT, GZMA, GZMK, ICOS, IFNB1, ITK, OR52N4, SELP, TXNRD3, ZAP70
Brain	AGBL4, APLNR, CNTN6, ERAS, GLDN, GPR75, GRIK4, HTR4, LHFPL4, SYNPR, ZFR2, ZNF488	ACTL6B, AGBL4, DCC, HTR4, KALRN, KCNAB1, MMD2, NAV3, NEUROG3, NSG2, PAQR6, RIPPLY2, SLC6A7, SLCO1A2, SYT4, UNC79, VN1R1, XKR7
Breast	ALX4	SCGB1D2, SPINK8
Cervix	SPINK6	
Colon		BCL2L15, CACNA1H, SPEG
Liver	A1CF, ABCG5, ABCG8, ACSM2A, ALB, ALDH1A1, APOA1, APOH, APOM, ASGR2, CYP3A7, CYP3A7-CYP3A51P, CYP4F3, FNDC4, HNMT, IDH1, ITIH2, LBX2, MMACHC, MPPED1, OTC, SERPINA10, SLC17A1, SLC22A9, SLCO1B3, SMLR1, TRIM40, UGT2A3	A1CF, ABCG5, ABCG8, ACSM2A, ACSM2B, ADH4, ADH6, AFM, AFP, AGXT, ALB, AMBP, ANGPTL8, ANKS4B, APOA1, APOB, APOC3, APOH, ARG1, ASGR1, ASGR2, AZGP1, C8A, C8B, CCL16, CHST13, CPN1, CREB3L3, CYP2D6, CYP8B1, F11, F7, FABP1, FGA, GJB1, HNF1A, HP, HPN, HPR, ITIH3, KLB, KNG1, MOGAT3, NEU4, NR1H4, NR1I3, ORM2, PLA2G12B, PON3, PRODH2, RBP4, RBP5, RIPPLY1, SAA4, SELENOP, SERPINA10, SERPINA4, SERPINA5, SERPINF2, SLC10A1, SLC10A5, SLC13A5, SLC17A1, SLC22A7, SLC22A9, SLC28A1, SLC38A3, SMLR1, SULT2A1, TAT, TFPI, TM4SF5, UGT2A3, UGT2B10, UNC5CL, UNC93A, UPB1, UROC1
Lung	ROS1, SPI1, VIPR1	MARCO, SFTPB
Prostate	CPA6, HOXB13	CWH43
Skin	AADACL3, C1orf74, FLG2, IL20, KRT72, KRT82, KRTAP9-9, LCE2A, SLC10A6	COMP, FAM25G, FLG, IL20, KRTAP9-9, LCE1F, LCE2A, SBSN, SERPINB12, STAC2, THEM5

## Discussions

TEs have been known to regulate gene expression at the steps of transcription, post-transcription, and translation level. In this study, we focused on TEs’ role in regulating transcription by offering sites of transcriptional regulation for other factors as part of promoters, enhancers, and repressors or insulators, and more specifically we aimed to examine the tissue specificity of TE-mediated gene regulation, particularly regarding the differences in the potential TE-regulated genes across tissues/cell lines. Different from prior systematic studies on this topic (Trizzino et al. 2018; Nikitin et al. 2019; Igolkina et al. 2019), we analyzed more than one type of regulatory sequences and for each cell line/tissue separately to capture more detailed features reflecting tissue-specificity in regulation.

### Regulatory sequences identified from different functional genomics methods

The three regulatory region datasets analyzed in this study are DHS (peak regions from DNase seq experiment), HA (peak regions of histone ChIP-seq experiment for active histone marks—H3K4me3, H3K27ac, H3K9ac, H3K79me2) and HR (peak regions of histone ChIP-seq experiment for repressive histone marks—H3K27me3, H3K9me3) generated by ENCODE (Feingold et al. 2004). Some of these datasets have been used by earlier studies for analysis of TEs in regulation using one data type, but not combining the histone marks and DHS data together (Jacques et al. 2013; Trizzino et al. 2018; Igolkina et al. 2019).

For functional data based on epigenetic marks, we selected only the ones that are widely accepted as markers for activation and repression of chromatin and avoided those with dual role (e.g., H3K36me3 (Chantalat et al. 2011)) or uncertainty for the role in gene activation and repression. For the repressive sites in genome, we considered the regions marked by H3K9me3 and H3K27me3 and for the active histone marks, we only considered H3K4me3, H3K27ac, H3K9ac, and H3K79me2, while some studies analyzing TEs in active chromatin considered H3K4me1, H3K36me3, and H3K4me3 (Trizzino et al. 2018) or H3K4me3, H3K9ac, and H3K27ac (Igolkina et al. 2019).

Among the three regulatory datasets under study, HR demarcates negative regulatory element/repressed regions while DHS and HA represent active regulatory elements, and the latter two provide complementary annotations for active regulatory elements (Hubbard and ENCODE Project Consortium 2011) with DHS capturing accessible genome regions regardless of histone marks and HA for active sites marked by activating histone marks. We observed only 16% overlap between the two regions, indicating that each of the two approaches identifies mostly a unique set of regulatory sequences (Fig. 1a). To further investigate differences between active regulatory sites captured by these two approaches, we examined the intersection of DHS and HA with (1) RNAPII binding sites, (2) transcription factor binding sites (TFBS), and (3) gene upstream regions (1.5 Kb upstream of TSS) within the same cell lines. Interestingly, DHS showed higher overlap with each of all these three regions, e.g., for RNAPII, it is 15% for DHS versus 9% for HA and almost nothing for HR (0.67%) (Table S11). For these reasons, we used DHS and HA for the analysis of TEs in active regulatory sites but treated them separately for them being largely different.

We examined the cell line specificity of the three sets of regulatory regions. By defining shared regions as those present in all 14 cell lines with the rest being cell line specific regions, a large fraction (> 95% in all three cases) was shown to be cell line specific. Further, we also sought to determine cell line unique regions as those identified in only one cell line for being highly cell line specific. By this definition, we found 59%, 39% and 61% being cell line unique regulatory sequences for DHS, HA and HR, respectively. On the other hand, it was interesting to observe the least proportion being shared in case of HR. Tissue specificity of both histone repressive marks we included in this study (H3K9me3 and H3K27me3) have been reported previously (Ninova et al. 2019; Zhu et al. 2012; Nicetto and Zaret 2019; Cai et al. 2021). Furthermore, Trizzino et al. (2018) in examining across-tissue variability of TE enrichment in active and repressed chromatin, showed higher variability of TE composition in repressed genomic regions, suggesting histone repressive regions being more variable across tissues.

Overall, DHS, HA, and HR regulatory sequences mostly showed to be cell line specific with a considerable portion locating into the gene-neighboring regions and each shown to be mostly unique group of regulatory sequences in the human genome by locations, average size, and rate being cell line specific and the TE profile.

### The pattern of TEs in regulatory regions

By determining the fraction of regulatory regions being TE-derived, we found it to be higher for cell line specific regulatory regions compared to shared regulatory regions and this trend was consistent for DHS, HA and HR (Fig. 2a, Tables 1, S12). This also coincides with the observation of Miao et al. in mouse (Miao et al. 2020) that among the TEs in accessible chromatin across 5 tissues, about half are present in only one tissue and only about 10% are common to all 5 tissues. TE activation is thus suggested as being strongly tissue-specific. Further, we examined the TE type composition and age profile in the regulatory regions. LTRs were shown to be enriched in DHS, which is in agreement with the findings from Jacques et al., reporting enrichment of LTRs in DHS of human normal, embryonic and cancerous cells (Jacques et al. 2013). However, our results showed that LTRs were also enriched in HR, while SINEs were enriched in HA, which agree with the results of Trizzino et al. in analyzing TEs in active and repressed chromatin (Trizzino et al. 2018). Our results further showed that the degree of these enrichment is even higher in the corresponding shared regulatory regions of the same type. However, the pattern of TE enrichment in gene-neighboring DHS, HA and HR was shown to be different with SINEs being the enriched TE type in all three types of regulatory regions, matching the previous findings that SINEs are more frequent in promoters than other regions (Kellner and Makałowski 2019) and SINE-derived TFBSs are more frequent in gene-neighboring sites compared to whole genome (Nikitin et al. 2018).

Via analyzing TEs’ age profile, we observed that TEs in regulatory regions tend to be older than the counterparts genome-wide (Figs. 3, 4), a pattern similar to what were observed for TFBS (Trizzino et al. 2018). We would agree with the authors of the study to reason that older TEs may have accumulated more TFBSs and are thus more likely to exapt for the regulatory role; they are also less likely to be transposition active/competent and are thus less likely to trigger the host genome for epigenetic suppression. In alignment with this, we also observed that TEs in cell line specific regulatory regions tend to be younger than those in the shared regions, indicating the special role of newly evolved TEs in tissue-specific gene regulation. As a way of confirming this, we used the human specific TE (HSTEs) data from our earlier study (Tang and Liang 2019) to examine ratio of HSTEs (vs all TEs) in regulatory regions that are present in (1) only one cell line, (2) two or more but not all 14 cell lines, and (3) all 14 cell lines. As expected, the ratio of HSTEs positively correlated with the cell line specificity of regulatory regions, which is quite consistent for all three types of regulatory sequences regardless of the genome context (Table S12). Furthermore, we observed that all TE types (except LINEs) in gene-neighboring regulatory regions are relatively younger, which could suggest that younger TEs’ contribution to regulatory novelty is more likely through gene-proximal regulation than gene-distal regulatory elements, but this requires further studies to confirm.

### Tissue-specific genes are enriched for TE-derived regulatory sites

Variation in the active/repressed states and regulatory activity of TEs across tissue types call for the comparative study of TE-regulated genes in different tissues. To the best of our knowledge, our study reported here is the first one addressing this issue by separately analyzing DHS, HA and HR regions in cell lines of 10 different tissue types. Comparison of genes (and associated biological processes) enriched with TE-derived regulatory sites, revealed interesting cell line specific patterns with some findings relating to tissue-specific functionalities, e.g., inferred TE-regulated genes in blood lymphocytes are enriched for immune related biological processes. TE-mediated lymphocyte-specific gene regulation has also been shown in a few other studies. For examples, Xie et al., identified instances of TEs near immune related genes being hypomethylated specifically in blood lymphocytes and harboring p300-binding sites (enhancer signature) in a lymphoblastoid cell line (Xie et al. 2013), while Trizzino et al. showed that active TEs in LCL harbor binding sites for PRDM1/Blimp-1 that is a prime factor in shaping lymphocyte differentiation (Trizzino et al. 2018). Besides immune functions of lymphocytes, we also found some other cases of TEs in regulating cell lines/tissue-specific processes (Table 3). Multiple metabolic processes GO terms and proteins inserting into mitochondrial membrane were found enriched only for liver cell line (HepG2). Moreover, some GO terms about mitochondrial RNA processing were shown to be enriched only for blood T-lineage cell line (DND-41) (Table 3), and elevated mitochondrial gene expression has been linked to T-cell activity (Kramerov and Vassetzky 2011). In analyzing TSGs, we also revealed some remarkably interesting findings (Tables 4, S9, S10). For examples, *ALB* (Albumin) gene was shown to be almost exclusively expressed in liver as known from literature (Cereghini et al. 1987), and it has 100% of neighboring DHS being TE-derived in the liver cell line and no TE-derived DHS in cell lines of other tissues. Similarly, *OTC* (Ornithine transcarbamylase), an important liver-elevated gene involved in urea cycle, also showed 100% of neighboring DHS being TE-derived, exclusively in liver cell line. These findings reflect a crucial role of TEs in promoting expression of nearby genes in a tissue-specific fashion.

## Summary and perspectives

In this study, by analyzing the pattern of TEs in three types of regulatory sequences in 14 human cell lines belonging to 10 different tissues, we demonstrated that while overall TEs are de-enriched in the regulatory sequences compared to their contribution to the genome, different type of regulatory sequences showed unique pattern of enrichment for TEs by type and age. By treating the data for each cell line separately, our study provides more detailed patterns for TEs’ contribution to regulatory sequences for tissue-specific regulation of genes conferring their tissue-specific expression. Overall, our result further enforces a pivotal role of TEs in tissue-specific gene regulation. Future directions of research on this topic may at least include similar analyses using new types of functional genomics data and/or from more tissue types and extending also to other organisms to see if similar patterns can be observed across species. Examining non-coding genes, orthologous profile of TE-regulated genes, and TE-derived alternative promoters in tissues producing tissue-specific transcript isoforms would also be interesting areas to explore. Certainly, experimental verification is needed to validate the roles of TEs in tissue-specific gene regulation identified via bioinformatics analyses. Many of the TSGs identified in this study that show TE-derived active regulatory sequences unique to a single cell line would certainly be very interesting cases to start with follow-up experimental studies.

## Supplementary Information

Below is the link to the electronic supplementary material.

Supplementary file1 (PPTX 815 KB)

Supplementary file2 (XLSX 7225 KB)

## References

[CR1] Ando H, Ichihashi M, Hearing V (2009) Role of the ubiquitin proteasome system in regulating skin pigmentation. Int J Mol Sci 10:4428–4434. 10.3390/ijms10104428 20057953 10.3390/ijms10104428 PMC2790116

[CR2] Ayarpadikannan S, Kim H-S (2014) The impact of transposable elements in genome evolution and genetic instability and their implications in various diseases. Genom Inform 12:98. 10.5808/gi.2014.12.3.98 10.5808/GI.2014.12.3.98 PMC4196381 25317108

[CR3] Balachandran P, Walawalkar IA, Flores JI, Dayton JN, Audano PA, Beck CR (2022) Transposable element-mediated rearrangements are prevalent in human genomes. Nat Commun 13:7115. 10.1038/s41467-022-34810-8 36402840 10.1038/s41467-022-34810-8 PMC9675761

[CR4] Bediaga NG, Coughlan HD, Johanson TM, Garnham AL, Naselli G, Schröder J, Fearnley LG, Bandala-Sanchez E, Allan RS, Smyth GK et al (2021) Multi-level remodelling of chromatin underlying activation of human T cells. Sci Rep 11:528. 10.1038/s41598-020-80165-9 33436846 10.1038/s41598-020-80165-9 PMC7804404

[CR5] Brini AT, Lee GM, Kinet JP (1993) Involvement of Alu sequences in the cell-specific regulation of transcription of the γ chain of Fc and T cell receptors. J Biol Chem 268:1355–1361 8419337

[CR6] Cai Y, Zhang Y, Loh YP, Tng JQ, Lim MC, Cao Z, Raju A, Lieberman Aiden E, Li S, Manikandan L et al (2021) H3K27me3-rich genomic regions can function as silencers to repress gene expression via chromatin interactions. Nat Commun 12:719. 10.1038/s41467-021-20940-y 33514712 10.1038/s41467-021-20940-y PMC7846766

[CR7] Cereghini S, Raymondjean M, Carranca AG, Herbomel P, Yaniv M (1987) Factors involved in control of tissue-specific expression of albumin gene. Cell 50:627–638. 10.1016/0092-8674(87)90036-5 3607880 10.1016/0092-8674(87)90036-5

[CR8] Chantalat S, Depaux A, Héry P, Barral S, Thuret J-Y, Dimitrov S, Gérard M (2011) Histone H3 trimethylation at lysine 36 is associated with constitutive and facultative heterochromatin. Genome Res 21:1426–1437. 10.1101/gr.118091.110 21803857 10.1101/gr.118091.110 PMC3166828

[CR9] Cordaux R, Batzer MA (2009) The impact of retrotransposons on human genome evolution. Nat Rev Genet 10:691–703. 10.1038/nrg2640 19763152 10.1038/nrg2640 PMC2884099

[CR10] Creyghton MP, Cheng AW, Welstead GG, Kooistra T, Carey BW, Steine EJ, Hanna J, Lodato MA, Frampton GM, Sharp PA et al (2010) Histone H3K27ac separates active from poised enhancers and predicts developmental state. Proc Natl Acad Sci 107:21931–21936. 10.1073/pnas.1016071107 21106759 10.1073/pnas.1016071107 PMC3003124

[CR11] D’Urso A, Brickner JH (2014) Mechanisms of epigenetic memory. Trends Genet 30:230–236. 10.1016/j.tig.2014.04.004 24780085 10.1016/j.tig.2014.04.004 PMC4072033

[CR12] de Koning APJ, Gu W, Castoe TA, Batzer MA, Pollock DD (2011) Repetitive elements may comprise over two-thirds of the human genome. PLoS Genet 7:e1002384. 10.1371/journal.pgen.1002384 22144907 10.1371/journal.pgen.1002384 PMC3228813

[CR13] Etchegaray E, Baas D, Naville M, Haftek-Terreau Z, Volff JN (2022) The neurodevelopmental gene MSANTD2 B elongs to a gene family formed by recurrent molecular domestication of harbinger transposons at the base of vertebrates. Mol Biol Evol. 10.1093/molbev/msac173 35980103 10.1093/molbev/msac173 PMC9392472

[CR14] Feingold EA et al (2004) The ENCODE (ENCyclopedia Of DNA elements) project. Science (80-) 306:636–640. 10.1126/science.1105136 10.1126/science.1105136 15499007

[CR15] Franchini LF, López-Leal R, Nasif S, Beati P, Gelman DM, Low MJ, De Souza FJS, Rubinstein M (2011) Convergent evolution of two mammalian neuronal enhancers by sequential exaptation of unrelated retroposons. Proc Natl Acad Sci USA 108:15270–15275. 10.1073/pnas.1104997108 21876128 10.1073/pnas.1104997108 PMC3174587

[CR16] Gerondakis S, Siebenlist U (2010) Roles of the NF- B pathway in lymphocyte development and function. Cold Spring Harb Perspect Biol 2:a000182–a000182. 10.1101/cshperspect.a000182 20452952 10.1101/cshperspect.a000182 PMC2857169

[CR17] Hambor JE, Mennone J, Coon ME, Hanke JH, Kavathas P (1993) Identification and characterization of an Alu-containing, T-cell-specific enhancer located in the last intron of the human CD8 alpha gene. Mol Cell Biol 13:7056–7070. 10.1128/mcb.13.11.7056 8413295 10.1128/mcb.13.11.7056-7070.1993 PMC364767

[CR18] Harrow J, Frankish A, Gonzalez JM, Tapanari E, Diekhans M, Kokocinski F, Aken BL, Barrell D, Zadissa A, Searle S et al (2012) GENCODE: the reference human genome annotation for the ENCODE project. Genome Res 22:1760–1774. 10.1101/gr.135350.111 22955987 10.1101/gr.135350.111 PMC3431492

[CR19] Hu Q, Xie Y, Ge Y, Nie X, Tao J, Zhao Y (2018) Resting T cells are hypersensitive to DNA damage due to defective DNA repair pathway. Cell Death Dis 9:662. 10.1038/s41419-018-0649-z 29855463 10.1038/s41419-018-0649-z PMC5981309

[CR20] Hubbard T, ENCODE Project Consortium (2011) A user’s guide to the encyclopedia of DNA elements (ENCODE). PLoS Biol 9:e1001046. 10.1371/journal.pbio.1001046 21526222 10.1371/journal.pbio.1001046 PMC3079585

[CR21] Hublitz P, Albert M, Hfmpeters A, Hublitz P, Albert M, Peters AHFM (2009) Mechanisms of transcriptional repression by histone lysine methylation. Int J Dev Biol 53:335–354. 10.1387/ijdb.082717ph 19412890 10.1387/ijdb.082717ph

[CR22] Igolkina AA, Zinkevich A, Karandasheva KO, Popov AA, Selifanova MV, Nikolaeva D, Tkachev V, Penzar D, Nikitin DM, Buzdin A (2019) H3K4me3, H3K9ac, H3K27ac, H3K27me3 and H3K9me3 histone tags suggest distinct regulatory evolution of open and condensed chromatin landmarks. Cells 8:1034. 10.3390/cells8091034 31491936 10.3390/cells8091034 PMC6770625

[CR23] Jacques PÉ, Jeyakani J, Bourque G (2013) The majority of primate-specific regulatory sequences are derived from transposable elements. PLoS Genet. 10.1371/journal.pgen.1003504 23675311 10.1371/journal.pgen.1003504 PMC3649963

[CR24] Jiang T, Zhou Z-M, Ling Z-Q, Zhang Q, Wu Z-Z, Yang J-W, Yang S-Y, Yang B, Huang L-S (2024) Pig H3K4me3, H3K27ac, and gene expression profiles reveal reproductive tissue-specific activity of transposable elements. Zool Res 45:138–151. 10.24272/j.issn.2095-8137.2023.060 38155423 10.24272/j.issn.2095-8137.2023.060 PMC10839656

[CR25] Kaimal V, Bardes EE, Tabar SC, Jegga AG, Aronow BJ (2010) ToppCluster: a multiple gene list feature analyzer for comparative enrichment clustering and network-based dissection of biological systems. Nucleic Acids Res 38:W96–W102. 10.1093/nar/gkq418 20484371 10.1093/nar/gkq418 PMC2896202

[CR26] Karolchik D (2003) The UCSC genome browser database. Nucleic Acids Res 31:51–54. 10.1093/nar/gkg129 12519945 10.1093/nar/gkg129 PMC165576

[CR27] Kehrl JH (2004) G-protein-coupled receptor signaling, RGS proteins, and lymphocyte function. Crit Rev Immunol 24:16. 10.1615/CritRevImmunol.v24.i6.20 10.1615/critrevimmunol.v24.i6.20 15777161

[CR28] Kellner M, Makałowski W (2019) Transposable elements significantly contributed to the core promoters in the human genome. Sci China Life Sci 62:489–497. 10.1007/s11427-018-9449-0 30915629 10.1007/s11427-018-9449-0

[CR29] Kim DS, Hahn Y (2011) Identification of human-specific transcript variants induced by DNA insertions in the human genome. Bioinformatics 27:14–21. 10.1093/bioinformatics/btq612 21037245 10.1093/bioinformatics/btq612

[CR30] Kramerov DA, Vassetzky NS (2011) Origin and evolution of SINEs in eukaryotic genomes. Heredity (edinb) 107:487–495. 10.1038/hdy.2011.43 21673742 10.1038/hdy.2011.43 PMC3242629

[CR31] Lander ES, Linton LM, Birren B, Nusbaum C, Zody MC, Baldwin J, Devon K, Dewar K, Doyle M, FitzHugh W et al (2001) Initial sequencing and analysis of the human genome. Nature 409:860–921. 10.1038/35057062 11237011 10.1038/35057062

[CR32] Liu N, Lee CH, Swigut T, Grow E, Gu B, Bassik MC, Wysocka J (2018) Selective silencing of euchromatic L1s revealed by genome-wide screens for L1 regulators. Nature 553:228–232. 10.1038/nature25179 29211708 10.1038/nature25179 PMC5774979

[CR33] Markiewicz E, Idowu OC (2019) DNA damage in human skin and the capacities of natural compounds to modulate the bystander signalling. Open Biol 9:190208. 10.1098/rsob.190208 31847786 10.1098/rsob.190208 PMC6936251

[CR34] Miao B, Fu S, Lyu C, Gontarz P, Wang T, Zhang B (2020) Tissue-specific usage of transposable element-derived promoters in mouse development. Genome Biol 21:255. 10.1186/s13059-020-02164-3 32988383 10.1186/s13059-020-02164-3 PMC7520981

[CR35] Nicetto D, Zaret KS (2019) Role of H3K9me3 heterochromatin in cell identity establishment and maintenance. Curr Opin Genet Dev 55:1–10. 10.1016/j.gde.2019.04.013 31103921 10.1016/j.gde.2019.04.013 PMC6759373

[CR36] Nikitin D, Penzar D, Garazha A, Sorokin M, Tkachev V, Borisov N, Poltorak A, Prassolov V, Buzdin AA (2018) Profiling of human molecular pathways affected by retrotransposons at the level of regulation by transcription factor proteins. Front Immunol 9:1–14. 10.3389/fimmu.2018.00030 29441061 10.3389/fimmu.2018.00030 PMC5797644

[CR37] Nikitin D, Garazha A, Sorokin M, Penzar D, Tkachev V, Markov A, Gaifullin N, Borger P, Poltorak A, Buzdin A (2019) Retroelement-linked transcription factor binding patterns point to quickly developing molecular pathways in human evolution. Cells. 10.3390/cells8020130 30736359 10.3390/cells8020130 PMC6406739

[CR38] Ninova M, Fejes Tóth K, Aravin AA (2019) The control of gene expression and cell identity by H3K9 trimethylation. Development. 10.1242/dev.181180 31540910 10.1242/dev.181180 PMC6803365

[CR39] Nishimura D (2000) RepeatMasker. Biotech Softw Internet Rep 1:36–39. 10.1089/152791600319259

[CR40] Pontis J, Planet E, Offner S, Turelli P, Duc J, Coudray A, Theunissen TW, Jaenisch R, Trono D (2019) Hominoid-specific transposable elements and KZFPs facilitate human embryonic genome activation and control transcription in naive human ESCs. Cell Stem Cell 24:724-735.e5. 10.1016/j.stem.2019.03.012 31006620 10.1016/j.stem.2019.03.012 PMC6509360

[CR41] Pontis J, Pulver C, Playfoot CJ, Planet E, Grun D, Offner S, Duc J, Manfrin A, Lutolf MP, Trono D (2022) Primate-specific transposable elements shape transcriptional networks during human development. Nat Commun 13:7178. 10.1038/s41467-022-34800-w 36418324 10.1038/s41467-022-34800-w PMC9684439

[CR42] Quinlan AR, Hall IM (2010) BEDTools: a flexible suite of utilities for comparing genomic features. Bioinformatics 26:841–842. 10.1093/bioinformatics/btq033 20110278 10.1093/bioinformatics/btq033 PMC2832824

[CR43] Rodriguez JM, Carro A, Valencia A, Tress ML (2015) APPRIS WebServer and WebServices. Nucleic Acids Res 43:W455–W459. 10.1093/nar/gkv512 25990727 10.1093/nar/gkv512 PMC4489225

[CR44] Roy AM, West NC, Rao A, Adhikari P, Alemán C, Barnes AP, Deininger PL (2000) Upstream flanking sequences and transcription of SINEs. J Mol Biol 302:17–25. 10.1006/jmbi.2000.4027 10964558 10.1006/jmbi.2000.4027

[CR45] SanMiguel P, Tikhonov A, Jin Y-K, Motchoulskaia N, Zakharov D, Melake-Berhan A, Springer PS, Edwards KJ, Lee M, Avramova Z et al (1996) Nested retrotransposons in the intergenic regions of the maize genome. Science (80-). 274:765–768. 10.1126/science.274.5288.765 10.1126/science.274.5288.765 8864112

[CR46] Swergold GD (1990) Identification, characterization, and cell specificity of a human LINE-1 promoter. Mol Cell Biol 10:6718–6729. 10.1128/mcb.10.12.6718 1701022 10.1128/mcb.10.12.6718-6729.1990 PMC362950

[CR47] Tang W, Liang P (2019) Comparative genomics analysis reveals high levels of differential retrotransposition among primates from the hominidae and the cercopithecidae families. Genome Biol Evol 11:3309–3325. 10.1093/gbe/evz234 31651947 10.1093/gbe/evz234 PMC6934888

[CR48] Tang W, Mun S, Joshi A, Han K, Liang P (2018) Mobile elements contribute to the uniqueness of human genome with 15,000 human-specific insertions and 14 Mbp sequence increase. DNA Res 25:521–533 30052927 10.1093/dnares/dsy022 PMC6191304

[CR49] Thul PJ, Lindskog C (2018) The human protein atlas: a spatial map of the human proteome. Protein Sci 27:233–244. 10.1002/pro.3307 28940711 10.1002/pro.3307 PMC5734309

[CR50] Trizzino M, Kapusta A, Brown CD (2018) Transposable elements generate regulatory novelty in a tissue-specific fashion. BMC Genom 19:1–12. 10.1186/s12864-018-4850-3 10.1186/s12864-018-4850-3 PMC6006921 29914366

[CR51] van Regenmortel MH, Mahy BW (eds) (2010) Desk encyclopedia of general virology. Academic Press, Cambridge

[CR52] Wysocka J, Swigut T, Xiao H, Milne TA, Kwon SY, Landry J, Kauer M, Tackett AJ, Chait BT, Badenhorst P et al (2006) A PHD finger of NURF couples histone H3 lysine 4 trimethylation with chromatin remodelling. Nature 442:86–90. 10.1038/nature04815 16728976 10.1038/nature04815

[CR53] Xie M, Hong C, Zhang B, Lowdon RF, Xing X, Li D, Zhou X, Lee HJ, Maire CL, Ligon KL et al (2013) DNA hypomethylation within specific transposable element families associates with tissue-specific enhancer landscape. Nat Genet 45:836–841. 10.1038/ng.2649 23708189 10.1038/ng.2649 PMC3695047

[CR54] Zattera ML, Bruschi DP (2022) Transposable elements as a source of novel repetitive DNA in the eukaryote genome. Cells 11:3373. 10.3390/cells11213373 36359770 10.3390/cells11213373 PMC9659126

[CR55] Zhu Y, van Essen D, Saccani S (2012) Cell-type-specific control of enhancer activity by H3K9 trimethylation. Mol Cell 46:408–423. 10.1016/j.molcel.2012.05.011 22633489 10.1016/j.molcel.2012.05.011

