# Supplementary material for: Transposable elements contribute to tissue-specific gene regulation in humans
Source: Genes Genomics. 2024 Aug 1;46(11):1327–43. doi: 10.1007/s13258-024-01550-6 (PMC11602805; doi:10.1007/s13258-024-01550-6)
Supplement: Supplementary file 1 — Supplementary file1 (PPTX 815 KB) [file 13258_2024_1550_MOESM1_ESM.pptx]

## Slide 1
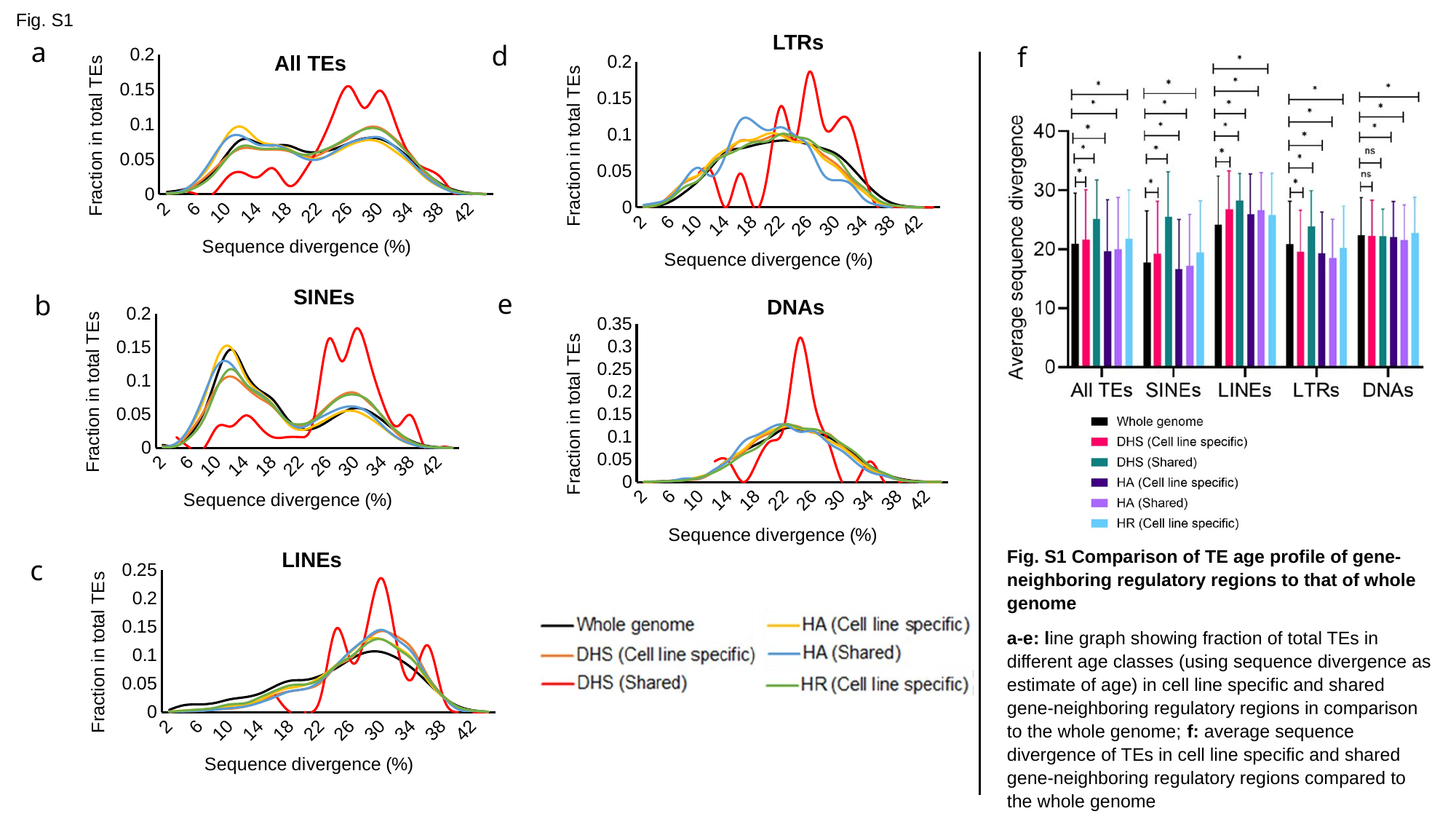

Fig. S1
LTRs
a
d
f
### Chart
| Category | Whole genome | DHS (Cell line specific) | DHS (shared) | HA (Cell line specific) | HA (Shared) | HR (Cell line specific) |
|---|---|---|---|---|---|---|
| 2 | 0.0033400866425189885 | 0.000817938794238519 | None | 0.0018617341411547566 | 0.001384234299868862 | 0.0008844938085433402 |
| 4 | 0.006434034625358141 | 0.0035311016239077523 | 0.006172839506172839 | 0.0047987802431488985 | 0.00513623779688183 | 0.0028564800046399674 |
| 6 | 0.014517690120195216 | 0.015421138730399393 | 0.0 | 0.02039882839144565 | 0.02141920442954976 | 0.010990923063538556 |
| 8 | 0.031113337978104564 | 0.033854686190799184 | 0.0 | 0.04724952854792762 | 0.051872359026664726 | 0.02692631151581939 |
| 10 | 0.059086323030580645 | 0.054343055500139646 | 0.024691358024691357 | 0.0853990290093488 | 0.080285589392394 | 0.052852130035089755 |
| 12 | 0.07887150877652192 | 0.06603359533974384 | 0.030864197530864196 | 0.09578301167596197 | 0.08265335858953811 | 0.06932401473189688 |
| 14 | 0.07314295759015925 | 0.064617164744843 | 0.024691358024691357 | 0.07747863419331541 | 0.07088736704065278 | 0.06598903807673347 |
| 16 | 0.069883705364112 | 0.06383912540398197 | 0.037037037037037035 | 0.07101071299602776 | 0.07030453154597115 | 0.06524954325319723 |
| 18 | 0.06967648697902967 | 0.061764353828352554 | 0.012345679012345678 | 0.06516069494041649 | 0.06360192335713245 | 0.06324855726009918 |
| 20 | 0.06175792187973806 | 0.05444280413358337 | 0.030864197530864196 | 0.05328411507442924 | 0.051835931808247124 | 0.056607603746773774 |
| 22 | 0.06057491661010396 | 0.05625822926225911 | 0.06790123456790123 | 0.05117361473337881 | 0.04994171645053184 | 0.06087057390598266 |
| 24 | 0.06474515987892801 | 0.06862705980928062 | 0.11728395061728394 | 0.05876499618825984 | 0.060359900917965906 | 0.06965751239741322 |
| 26 | 0.07320262108803124 | 0.08005825320193113 | 0.15432098765432098 | 0.06998354933194238 | 0.07318228180096167 | 0.08250442246904272 |
| 28 | 0.08001815994351 | 0.09384351434385349 | 0.12345679012345678 | 0.07701320065802672 | 0.07952061780562436 | 0.09288634979555144 |
| 30 | 0.0793909447490348 | 0.0950604476718669 | 0.14814814814814814 | 0.07483047787184528 | 0.08137840594492204 | 0.0929733491865557 |
| 32 | 0.06854737132037673 | 0.07961935921477875 | 0.10493827160493827 | 0.06158969626449464 | 0.06815532565933265 | 0.07769045616680684 |
| 34 | 0.051046720154244045 | 0.05821330247775605 | 0.05555555555555555 | 0.04476186654897083 | 0.047756083345475736 | 0.05530261288170983 |
| 36 | 0.03153611480711155 | 0.030642780193911343 | 0.037037037037037035 | 0.024290815712394175 | 0.026081888387002768 | 0.03262477162659862 |
| 38 | 0.015357683738980965 | 0.013685512508478635 | 0.024691358024691357 | 0.010552501705252177 | 0.011073874398950896 | 0.014427399008206943 |
| 40 | 0.005691769388391408 | 0.004009895064437617 | 0.0 | 0.0034586526501625007 | 0.002149205886638496 | 0.004509468433720964 |
| 42 | 0.0017024412421469792 | 0.001037385787814707 | 0.0 | 0.0009870400834570477 | 0.0008013988051872359 | 0.001290490966563234 |
| 44 | 0.00036204409282186104 | 0.0002792961736424211 | 0.0 | 0.00016851903863900815 | 0.0002185633105056098 | 0.0003334976655163414 |All TEs
### Chart
| Category | Whole genome | DHS (Cell line specific) | DHS (shared) | HA (Cell line specific) | HA (Shared) | HR (Cell line specific) |
|---|---|---|---|---|---|---|
| 2 | 0.0007587527067300563 | 0.0004700720777185835 | None | 0.0005683971201212581 | 0.0032414910858995136 | 0.0006082725060827251 |
| 4 | 0.002015770835309252 | 0.0034471952366029457 | None | 0.00407351269420235 | 0.006482982171799027 | 0.0031427412814274127 |
| 6 | 0.008370324754173474 | 0.017862738953306173 | None | 0.015915119363395226 | 0.014586709886547812 | 0.012368207623682076 |
| 8 | 0.02103134263161621 | 0.03462864305860232 | None | 0.03618794998105343 | 0.03808752025931929 | 0.028183292781832927 |
| 10 | 0.036791491283026785 | 0.04450015669069257 | 0.046511627906976744 | 0.04499810534293293 | 0.05429497568881685 | 0.03751013787510138 |
| 12 | 0.05741139758775416 | 0.06330303979943591 | 0.046511627906976744 | 0.06574460022735885 | 0.04294975688816856 | 0.06032035685320357 |
| 14 | 0.076198542072704 | 0.0742713882795362 | 0.0 | 0.07985979537703676 | 0.07455429497568881 | 0.07208029197080291 |
| 16 | 0.0810649788871716 | 0.09150736446255092 | 0.046511627906976744 | 0.0907540735126942 | 0.11912479740680713 | 0.08100162206001622 |
| 18 | 0.08550528522021862 | 0.09088060169225948 | 0.0 | 0.09350132625994695 | 0.11750405186385737 | 0.08961881589618816 |
| 20 | 0.0895314835619165 | 0.09573801316201817 | 0.046511627906976744 | 0.10126942023493747 | 0.10615883306320907 | 0.09113949716139497 |
| 22 | 0.09202681816785266 | 0.09996866186148542 | 0.13953488372093023 | 0.09899583175445244 | 0.11021069692058347 | 0.10107461476074615 |
| 24 | 0.09088735327552742 | 0.09260419931056095 | 0.09302325581395349 | 0.08990147783251232 | 0.09886547811993517 | 0.09712084347120843 |
| 26 | 0.08663005595801213 | 0.08759009714822939 | 0.18604651162790697 | 0.08781735505873436 | 0.08346839546191248 | 0.09296431467964315 |
| 28 | 0.08108501637062399 | 0.07270448135380758 | 0.11627906976744186 | 0.06830238726790451 | 0.046191247974068074 | 0.07887266828872669 |
| 30 | 0.0732757411531171 | 0.05907239109996866 | 0.11627906976744186 | 0.055039787798408485 | 0.03727714748784441 | 0.06914030819140309 |
| 32 | 0.056520397490238405 | 0.039486054528361014 | 0.11627906976744186 | 0.03552482000757863 | 0.03241491085899514 | 0.043694241686942414 |
| 34 | 0.03555317480566981 | 0.021153243497336258 | 0.046511627906976744 | 0.020841227737779463 | 0.011345218800648298 | 0.026358475263584754 |
| 36 | 0.017321736528465918 | 0.007364462550924475 | 0.0 | 0.007294429708222812 | 0.0032414910858995136 | 0.010239253852392539 |
| 38 | 0.006008573371253157 | 0.0025070510811657787 | 0.0 | 0.0027472527472527475 | 0.0 | 0.0033454987834549877 |
| 40 | 0.0016644469587775532 | 0.0007834534628643058 | 0.0 | 0.000663129973474801 | 0.0 | 0.0011151662611516627 |
| 42 | 0.0003099130773967836 | 0.0 | 0.0 | 0.0 | 0.0 | 0.00010137875101378751 |
| 44 | 3.74033024444394e-05 | 0.00015669069257286117 | 0.0 | 0.0 | 0.0 | 0.0 |SINEs
e
b
DNAs
### Chart
| Category | Whole genome | DHS (Cell line specific) | DHS (shared) | HA (Cell line specific) | HA (Shared) | HR (Cell line specific) |
|---|---|---|---|---|---|---|
| 2 | 0.004598705085836377 | 0.0014089042750181145 | None | 0.0026090880884551376 | 0.0017354877318970677 | 0.0012672665061462425 |
| 4 | 0.004077241403136771 | 0.004790274535061589 | 0.016129032258064516 | 0.0060784700871576456 | 0.007241172950329144 | 0.00297807628944367 |
| 6 | 0.020497409415928286 | 0.024675952016745835 | 0.0 | 0.030759033086057597 | 0.03267504488330341 | 0.016537827905208465 |
| 8 | 0.05428836401835811 | 0.05534981080428307 | 0.0 | 0.073689109525287 | 0.07929383602633154 | 0.04384742111265999 |
| 10 | 0.11203695806373949 | 0.09306819096691088 | 0.03225806451612903 | 0.13653287450991453 | 0.12357869539198085 | 0.09241540996071473 |
| 12 | 0.14650052524240503 | 0.10627163674422349 | 0.03225806451612903 | 0.14812568752996927 | 0.12399760622381807 | 0.11741224179444937 |
| 14 | 0.11224802669721314 | 0.09113597938974317 | 0.04838709677419355 | 0.1061405240741263 | 0.09605026929982047 | 0.09583702952730959 |
| 16 | 0.08633030999558429 | 0.07479268979953305 | 0.03225806451612903 | 0.08364595379798606 | 0.08126870137642131 | 0.0793942466100621 |
| 18 | 0.07175523811888725 | 0.06118669994364383 | 0.016129032258064516 | 0.0648887259188221 | 0.06361460203470976 | 0.06298314535546826 |
| 20 | 0.044310377775066696 | 0.039489574108364865 | 0.016129032258064516 | 0.03776831297774518 | 0.040754039497307 | 0.04064757318464073 |
| 22 | 0.028874297022690686 | 0.0330488688511392 | 0.016129032258064516 | 0.026767833469664062 | 0.030460801915020947 | 0.03519832720821189 |
| 24 | 0.029388743078190404 | 0.04456162949843008 | 0.04838709677419355 | 0.03315657349166502 | 0.04021543985637343 | 0.0423267013052845 |
| 26 | 0.0401332701387007 | 0.0634409467836728 | 0.16129032258064516 | 0.0442134657151722 | 0.051406343506882106 | 0.061367380560131794 |
| 28 | 0.05274448603438205 | 0.07881813058529909 | 0.12903225806451613 | 0.05393055594731053 | 0.0599640933572711 | 0.07647953364592573 |
| 30 | 0.05935293158677188 | 0.08171644795105064 | 0.1774193548387097 | 0.05445237356500155 | 0.06128067025733094 | 0.07920415663414015 |
| 32 | 0.05392884557873292 | 0.06637951855728202 | 0.12903225806451613 | 0.04446732293458946 | 0.051226810293237585 | 0.06890761627170194 |
| 34 | 0.03973812374559914 | 0.04580951614201755 | 0.06451612903225806 | 0.029800016923814628 | 0.03189706762417714 | 0.04454441769104043 |
| 36 | 0.02325803599287009 | 0.021334836164560018 | 0.03225806451612903 | 0.014667306010774829 | 0.01538001196888091 | 0.02461665188189076 |
| 38 | 0.010741827973125727 | 0.009178004991546574 | 0.04838709677419355 | 0.00579640651002736 | 0.006044284859365649 | 0.009916360410594348 |
| 40 | 0.003830544816186751 | 0.0025762821028902667 | 0.0 | 0.0018898259667729106 | 0.0012567324955116697 | 0.0030414396147509823 |
| 42 | 0.0011325370665670523 | 0.0007648337492955478 | 0.0 | 0.0005077144388345133 | 0.00047875523638539794 | 0.0008870865543023698 |
| 44 | 0.00023320115002715283 | 0.00020127203928830206 | 0.0 | 0.00011282543085211407 | 0.00017953321364452425 | 0.0001900899759219364 |
### Chart
| Category | Whole genome | DHS (Cell line specific) | DHS (shared) | HA (Cell line specific) | HA (Shared) | HR (Cell line specific) |
|---|---|---|---|---|---|---|
| 2 | 0.00027850855662439616 | 0.000189717321191425 | None | 0.0002434077079107505 | None | 0.0004453681710213777 |
| 4 | 0.0014366232740985038 | 0.0009485866059571238 | None | 0.0007302231237322516 | 0.0007220216606498195 | 0.0011876484560570072 |
| 6 | 0.0030595868055068557 | 0.0022766078542970974 | None | 0.002758620689655172 | 0.002888086642599278 | 0.0019299287410926365 |
| 8 | 0.006087115072121695 | 0.00474293302978562 | None | 0.005598377281947261 | 0.007942238267148015 | 0.00430522565320665 |
| 10 | 0.010757643456952395 | 0.007588692847656991 | None | 0.012251521298174441 | 0.009025270758122744 | 0.010540380047505939 |
| 12 | 0.025899292107388092 | 0.024473534433693798 | 0.045454545454545456 | 0.02515212981744422 | 0.02779783393501805 | 0.02211995249406176 |
| 14 | 0.05036596825803918 | 0.048188199582621896 | 0.045454545454545456 | 0.05038539553752536 | 0.04837545126353791 | 0.03948931116389549 |
| 16 | 0.06784187927154985 | 0.06526275848985012 | 0.0 | 0.07026369168356998 | 0.08772563176895307 | 0.060570071258907364 |
| 18 | 0.08389519262172727 | 0.09409979131094669 | 0.045454545454545456 | 0.09849898580121703 | 0.10397111913357401 | 0.07586104513064133 |
| 20 | 0.10068384870774033 | 0.10927717700626068 | 0.09090909090909091 | 0.11375253549695741 | 0.12093862815884476 | 0.10495843230403801 |
| 22 | 0.11889309879840589 | 0.12312654145323468 | 0.13636363636363635 | 0.12267748478701826 | 0.1263537906137184 | 0.1278206650831354 |
| 24 | 0.11662094985443419 | 0.12160880288370328 | 0.3181818181818182 | 0.118052738336714 | 0.1111913357400722 | 0.11965558194774346 |
| 26 | 0.11046571038716697 | 0.10889774236387782 | 0.18181818181818182 | 0.10985801217038539 | 0.11227436823104693 | 0.11683491686460808 |
| 28 | 0.10002664866045399 | 0.107190286473155 | 0.09090909090909091 | 0.09241379310344827 | 0.08736462093862817 | 0.10525534441805226 |
| 30 | 0.08058715213980729 | 0.07911212293682413 | 0.0 | 0.07602434077079108 | 0.06859205776173286 | 0.08417458432304038 |
| 32 | 0.05787768465217487 | 0.05369000189717321 | 0.0 | 0.050953346855983774 | 0.04259927797833935 | 0.06279691211401425 |
| 34 | 0.03483560982353778 | 0.02504268639726807 | 0.045454545454545456 | 0.029127789046653144 | 0.022021660649819495 | 0.03399643705463183 |
| 36 | 0.018375553761167892 | 0.015367103016505406 | 0.0 | 0.014523326572008114 | 0.013718411552346571 | 0.019596199524940617 |
| 38 | 0.008016638381684957 | 0.006829823562891292 | 0.0 | 0.004543610547667343 | 0.005415162454873646 | 0.006383610451306413 |
| 40 | 0.0029113160631312777 | 0.001707455890722823 | 0.0 | 0.0016227180527383367 | 0.0010830324909747292 | 0.001336104513064133 |
| 42 | 0.0008715915261267073 | 0.00018971732119142478 | 0.0 | 0.00040567951318458417 | 0.0 | 0.0004453681710213777 |
| 44 | 0.00021238782015961145 | 0.00018971732119142478 | 0.0 | 0.00016227180527383366 | 0.0 | 0.0002969121140142518 |
Fig. S1 Comparison of TE age profile of gene-neighboring regulatory regions to that of whole genome
a-e: line graph showing fraction of total TEs in different age classes (using sequence divergence as estimate of age) in cell line specific and shared gene-neighboring regulatory regions in comparison to the whole genome; f: average sequence divergence of TEs in cell line specific and shared gene-neighboring regulatory regions compared to the whole genome
LINEs
c
### Chart
| Category | Whole genome | DHS (Cell line specific) | DHS (shared) | HA (Cell line specific) | HA (Shared) | HR (Cell line specific) |
|---|---|---|---|---|---|---|
| 2 | 0.003975637993420424 | 7.362143856290952e-05 | None | 0.0010434328942219902 | 0.0005962140408406618 | 0.000482392667631452 |
| 4 | 0.012324286735009685 | 0.0016196716483840095 | None | 0.0029346550149993477 | 0.001341481591891489 | 0.002701398938736131 |
| 6 | 0.01359154920608235 | 0.0022086431568872854 | None | 0.004597626190165645 | 0.002384856163362647 | 0.004582730342498794 |
| 8 | 0.01612989504010026 | 0.005153500699403666 | None | 0.0063584191991652534 | 0.004173498285884632 | 0.007718282682103232 |
| 10 | 0.022522247142928102 | 0.00611057940072149 | None | 0.010727794443719837 | 0.007005514979877776 | 0.013699951760733237 |
| 12 | 0.026322124110209805 | 0.010012515644555695 | None | 0.013988522238163558 | 0.009986585184081085 | 0.01587071876507477 |
| 14 | 0.0330035903647296 | 0.01818449532503865 | None | 0.021683839833050736 | 0.017141153674169025 | 0.026386878919440426 |
| 16 | 0.04600162769993772 | 0.03033203268791872 | 0.029411764705882353 | 0.035574540237380985 | 0.026978685348039946 | 0.03796430294259527 |
| 18 | 0.055343071513085915 | 0.03681071928145476 | 0.0 | 0.04284596321899048 | 0.03696527053212103 | 0.047226242161119154 |
| 20 | 0.05686569692430941 | 0.04115438415666642 | 0.0 | 0.0483892004695448 | 0.0409897153077955 | 0.048866377231066085 |
| 22 | 0.0645272219441462 | 0.05234484281822867 | 0.029411764705882353 | 0.06250815181948612 | 0.05589506632881204 | 0.05914134105161602 |
| 24 | 0.07759467214837287 | 0.08083633954207466 | 0.14705882352941177 | 0.08334420242598148 | 0.08242659114622149 | 0.0819585142305837 |
| 26 | 0.09406207930388445 | 0.09570787013178238 | 0.08823529411764706 | 0.10727794443719839 | 0.10895811596363095 | 0.09860106126386879 |
| 28 | 0.10541522268794648 | 0.12633438857395274 | 0.14705882352941177 | 0.12726620581713838 | 0.1307199284543151 | 0.12030873130728413 |
| 30 | 0.10562600855625054 | 0.14260472649635575 | 0.23529411764705882 | 0.12811399504369375 | 0.14473095841407066 | 0.12807525325615052 |
| 32 | 0.0949905560289216 | 0.13288669660605168 | 0.11764705882352941 | 0.11441893830703014 | 0.1275898047399016 | 0.11201157742402315 |
| 34 | 0.07700307072343493 | 0.1113892365456821 | 0.058823529411764705 | 0.09394156775792357 | 0.10463556416753615 | 0.09247467438494934 |
| 36 | 0.05233093508686798 | 0.06449238018110874 | 0.11764705882352941 | 0.05644319812182079 | 0.0617081532270085 | 0.05981669078630005 |
| 38 | 0.02763332683781715 | 0.029816682617978357 | 0.029411764705882353 | 0.026705360636494067 | 0.028022059919511105 | 0.02923299565846599 |
| 40 | 0.010710596734156353 | 0.009055436943237872 | 0.0 | 0.008803965044998044 | 0.0052168728573557905 | 0.009406657018813314 |
| 42 | 0.0033088923616550574 | 0.0023558860340131046 | 0.0 | 0.00267379679144385 | 0.002086749142942316 | 0.0027496382054992764 |
| 44 | 0.000717690856733112 | 0.0005153500699403667 | 0.0 | 0.0003586800573888092 | 0.00044716053063049635 | 0.000723589001447178 |

## Slide 2
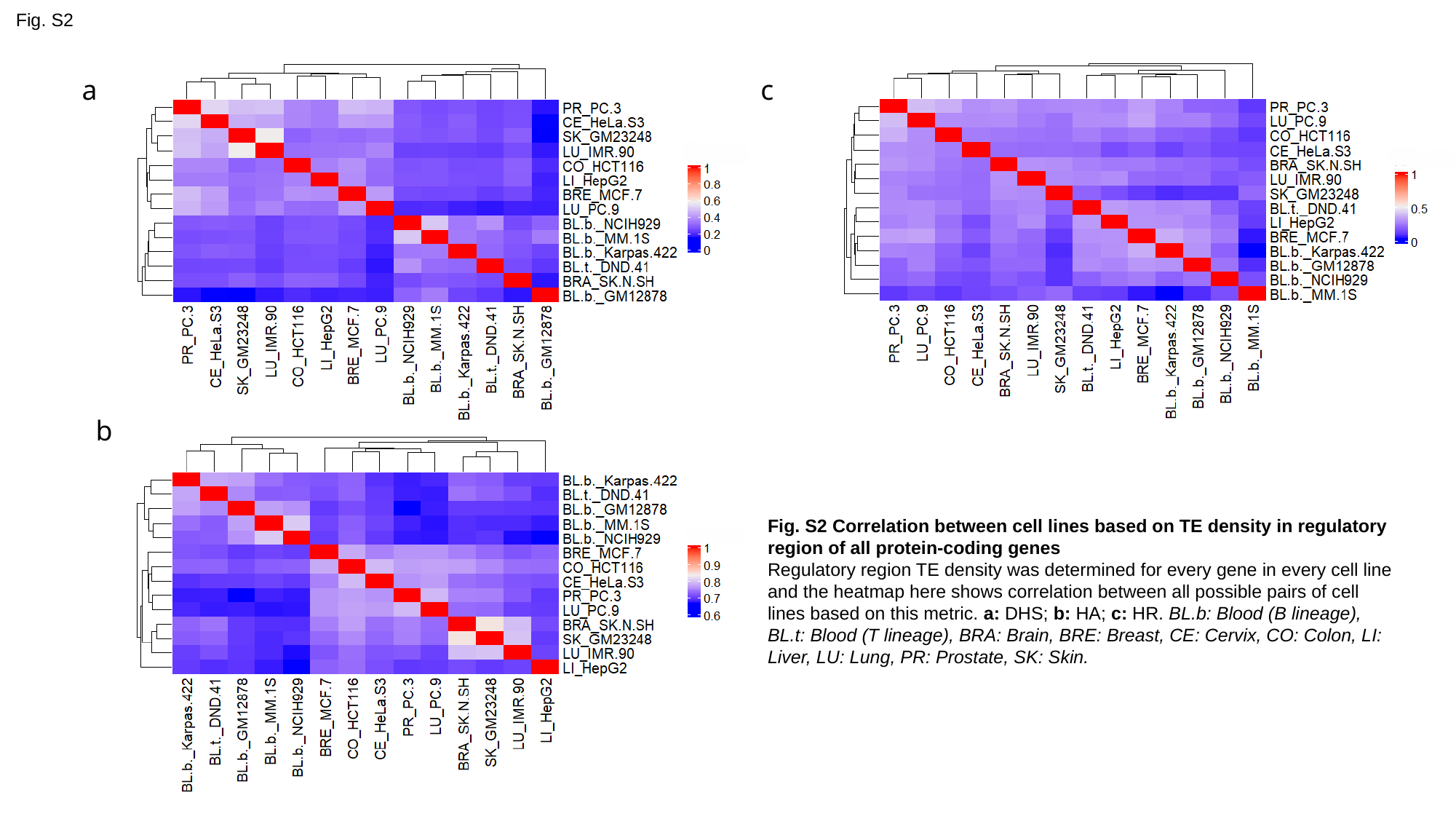

Fig. S2
a
c
b
Fig. S2 Correlation between cell lines based on TE density in regulatory region of all protein-coding genes
Regulatory region TE density was determined for every gene in every cell line and the heatmap here shows correlation between all possible pairs of cell lines based on this metric. a: DHS; b: HA; c: HR. BL.b: Blood (B lineage), BL.t: Blood (T lineage), BRA: Brain, BRE: Breast, CE: Cervix, CO: Colon, LI: Liver, LU: Lung, PR: Prostate, SK: Skin.
